# Static and Dynamic Responses of One-Dimensional Hexagonal Piezoelectric Quasicrystal Microbeams Resting on Elastic Foundations

**DOI:** 10.3390/ma19184020

**Published:** 2026-09-21

**Authors:** Shihan Zhang, Li Zhang, Lei Li, Bojie Liu

**Affiliations:** College of Mechanics and Aeronautics, Inner Mongolia University of Technology, Hohhot 010050, China; 15318659871@163.com (S.Z.); leillt@163.com (L.L.); 15540956149@163.com (B.L.)

**Keywords:** 1D hexagonal piezoelectric quasicrystal, modified couple stress theory, size effect, Winkler–Pasternak elastic foundation, static and dynamic responses

## Abstract

At the microscale, the interplay between size effects and boundary constraints strongly influences the mechanical behavior of quasicrystal structures. To elucidate this interplay, we investigate the static and dynamic responses of one-dimensional (1D) hexagonal piezoelectric quasicrystal Timoshenko microbeams. Our model integrates the modified couple stress theory with a Winkler–Pasternak two-parameter elastic foundation. Under open-circuit conditions, the electric potential is generated solely by mechanical deformation through piezoelectric coupling. Using Hamilton’s principle, we establish a non-classical beam theory framework for piezoelectric quasicrystals that simultaneously incorporates size effects, piezoelectric coupling, and foundation constraints. A normalized system contact stiffness is introduced to quantify the coupling between size effects and the foundation, providing a possible reference for evaluating foundation parameters. Fourier series solutions reveal that increasing the material length scale parameter markedly enhances both bending stiffness and the free vibration frequencies, particularly at small scales. The elastic foundation further suppresses static deformation and elevates all natural frequencies. Notably, the phason field exhibits a non-classical, non-monotonic response under foundation constraints. Increasing the Pasternak shear stiffness not only reduces the overall phason deflection but also triggers a qualitative reversal of the size sensitivity. The apparent critical Pasternak stiffness required for this transition decreases systematically as the Winkler spring stiffness increases, indicating a synergistic effect between the two foundation parameters. Detailed scanning around the critical interval further reveals a transitional regime in which the phason deflection first rises and then declines, signifying a competitive handover between two driving mechanisms within a narrow stiffness band. Unlike existing size-dependent quasicrystal beam models, the present formulation simultaneously incorporates a two-parameter elastic foundation and piezoelectric coupling in a Timoshenko microbeam, and further reveals a non-monotonic phason response governed by a critical energy partition ratio. These findings provide theoretical insight into the complex multi-field coupling in quasicrystals, and the open-circuit formulation may be particularly relevant for high-frequency or electrically insulated configurations.

## 1. Introduction

The first experimental observation of quasicrystalline structures was reported in melt-spun Al-Mn alloys [1]. These structures possess long-range quasiperiodic order yet lack translational symmetry, which challenges the fundamental assumptions of classical crystallography and has drawn intense research interest. Unlike conventional crystals, a complete mechanical description of quasicrystals requires two independent displacement fields: the phonon field, representing the displacement of lattice points, and the phason field, characterizing the local atomic rearrangements needed to restore quasiperiodicity under deformation. A distinctive feature of the phason field is that it carries no inertial mass. Structurally intermediate between crystals and glasses, quasicrystals combine properties seldom found in ordinary crystals, including high hardness, low friction, excellent wear resistance, non-stick surfaces, good thermal stability, and poor thermal conductivity [2,3,4,5]. Owing to these properties, quasicrystals show significant application potential in aerospace, defense, precision coating and other fields. Among them, one-dimensional hexagonal quasicrystals have become important subjects for theoretical and experimental investigations because of their relatively simple structure and mature preparation technology. In recent years, research on one-dimensional hexagonal quasicrystals has expanded to include functionally graded configurations [6], interfacial behaviors of quasicrystal films [7], and multi-field coupling problems under thermal and electrical loads [6,7], reflecting the growing interest in applying these materials to advanced multifunctional devices.

Advances in micro- and nanotechnologies have enabled quasicrystal microbeams and microplates to be employed in MEMS, sensors, energy harvesters, and similar devices. In materials engineering, these structures are closely related to quasicrystal films or coatings on elastic substrates, functionally graded quasicrystal laminates, and piezoelectric micro-devices, where substrate or foundation stiffness can significantly influence the overall mechanical and electromechanical behavior. When structural dimensions shrink to the micron or submicron scale, however, experiments reveal pronounced size effects that classical continuum mechanics cannot accurately capture [8,9]. Therefore, size effects must be considered when analyzing microscale quasicrystal structures. Moreover, in practical engineering, beams are often supported on elastic foundations, which can significantly alter their static and dynamic responses. It is thus necessary to establish a mechanical model of quasicrystal microbeams that simultaneously incorporates size effects, piezoelectric coupling, and foundation support, to reveal the underlying multi-physical coupling mechanism and to provide a theoretical reference for the mechanical analysis of quasicrystal microbeams on elastic foundations.

Early investigations of quasicrystal mechanics focused primarily on elasticity. Fan et al. [10] examined dislocations in 1D hexagonal quasicrystals, and subsequently Liu and Fan [11] extended that work to analyze the interaction between dislocations and cracks in the same material. Liu et al. [12] provided a systematic derivation and formulation of the fundamental framework for plane elasticity problems in one-dimensional quasicrystals. As the theory matured, Sladek et al. [13] further studied the bending problem of one-dimensional orthogonal quasicrystal plates. Yang et al. [14,15,16] obtained exact analytical solutions for simply supported layered quasicrystal plates using the pseudo-Stroh formalism. Chen et al. [17] derived exact solutions for the vibration and buckling of one-dimensional hexagonal quasicrystal laminated beams. With the rapid development of micro- and nano-devices, research on quasicrystal beams and plates has shifted toward size effects at small scales. Guo et al. [18,19] performed several pioneering studies employing both nonlocal elasticity and modified couple stress theory. They analyzed the three-dimensional buckling of coated quasicrystal composites and laminated plates embedded in elastic media, quantifying the influences of size effects and lamination parameters, and revealing the strengthening effect of the elastic foundation on structural stability. These works have advanced the understanding of quasicrystal structures, yet the coupled influence of piezoelectricity and a two-parameter elastic foundation in microbeams remains largely unexplored. Recently, the mechanical behavior of quasicrystal beams and plates has attracted increasing attention. Zhang et al. [7] investigated the interfacial behavior of one-dimensional hexagonal piezoelectric quasicrystal films based on beam theory. Luo et al. [6] performed interfacial analysis of functionally graded piezoelectric quasicrystal films on temperature-dependent substrates. Zhang et al. [20] studied the axisymmetric contact problem of one-dimensional hexagonal quasicrystals based on couple stress theory. Vibration and bending of one-dimensional quasicrystal layered beams interacting with elastic media have been systematically analyzed [21], indicating that Winkler stiffness and shear modulus significantly enhance the natural frequencies and buckling capacity. Fracture studies have addressed transient impact fracture of functionally graded piezoelectric quasicrystal strips [22] and the electric polarization saturation model for penny-shaped cracks in one-dimensional hexagonal piezoelectric quasicrystals [23]. These developments indicate a rapidly growing research interest in the multi-field coupling behavior of piezoelectric quasicrystals.

Nevertheless, most of the above studies have focused on elastic quasicrystal structures, and the simultaneous incorporation of piezoelectric coupling and a two-parameter elastic foundation in a microbeam framework remains relatively unexplored. A key concern is the electrical conductivity of quasicrystalline alloys such as Al-Ni-Co. Under quasi-static conditions, free charges in metallic quasicrystals may screen the piezoelectric signal, limiting direct device applications. However, this does not rule out the piezoelectric effect in quasicrystals. Theoretically, the piezoelectric effect in quasicrystals is well established and originates from the combined contributions of the phonon and phason fields [24,25]. First-principles calculations have predicted material parameters of 1D hexagonal piezoelectric quasicrystals [26], supporting the inclusion of piezoelectric coupling in such materials. The concept of “piezoelectric metals” further indicates that metallicity and piezoelectricity are not mutually exclusive [27]. More importantly, under high-frequency dynamic conditions, the shielding effect of conductivity is weakened. Metal electrodes such as Al, Ag, Au, and Cu are widely used in high-frequency piezoelectric devices, and their dissipation cannot be explained solely by direct-current conductivity [28,29]. Experiments also show that near a resonant piezoelectric element, the piezoelectric response can still be measured, and the interference decreases with increasing frequency [30]. Recent theoretical and review works have systematically described the symmetry and unique electromechanical coupling behavior of piezoelectric quasicrystals [31,32], and PFM has been used to characterize quasicrystal samples [33]. Together, these findings provide a physical basis and experimental support for establishing a piezoelectric quasicrystal microbeam model that considers both size effects and foundation constraints. To this end, the modified couple stress theory provides a suitable framework for capturing size effects at the microscale.

As micro/nanotechnology continues to advance, quasicrystal microstructure devices have demonstrated considerable potential; however, the size effect at the microscale has become an inevitable theoretical challenge. Lam et al. [8] experimentally verified the size effect in microstructures, while Toupin [34] proposed the classical couple stress theory, providing a theoretical foundation for explaining this phenomenon. In the same period, Mindlin et al. [35] further refined the mechanical effects of couple stress within the linear elasticity framework advanced by Toupin. Yang et al. [36] later streamlined the classical couple stress theory into a more practical form, now widely known as the modified couple stress theory. Within a third-order shear deformation theory, Gao and Zhang [37] simultaneously introduced the modified couple stress theory and surface elasticity theory to accurately describe the mechanical behavior of micro/nano-scale beams. Guo et al. [38,39] combined this theoretical framework with surface effects and successfully characterized the mechanical behavior of micro/nano-sized structures. Building on these developments, Li et al. [40] and Zhang et al. [41] further extended the size effect theories to piezoelectric quasicrystals and magnetoelectroelastic materials.

In practical engineering, beam and plate structures are often supported on elastic foundations. To describe such interactions, Ting [42] established the mathematical formulation of the classical Winkler and Pasternak two-parameter foundation models. Limkatanyu et al. [43] derived the exact stiffness matrix for beam elements on elastic foundations. Subsequently, Van Thom et al. [44] and Khajeansari et al. [45] investigated the mechanical responses of functionally graded materials and nanobeams resting on foundations, respectively. Limkatanyu et al. [46] further combined the nonlocal elasticity model with the foundation model to establish a unified theoretical framework for the analysis of nano-scale foundation-structure systems. The Winkler–Pasternak model extends the classic Winkler foundation by incorporating a Pasternak shear layer that captures the foundation’s continuous shear behavior, thereby providing a more realistic description of its overall mechanical response.

This paper aims to reveal the coupling between size effects and foundation constraints in piezoelectric quasicrystal microbeams. A Timoshenko microbeam model is developed by combining the modified couple stress theory with a Winkler–Pasternak elastic foundation, thus integrating size effects, piezoelectric coupling, and foundation support in one framework. The simultaneous consideration of a two-parameter foundation and piezoelectric quasicrystal coupling has received limited attention, and the resulting size–foundation interaction remains insufficiently quantified. To partly fill this gap, a normalized system contact stiffness is introduced to quantify the stiffness change induced by the size effect under different foundation parameter combinations; it may serve as a possible reference for evaluating the foundation effect under the present idealized assumptions. Furthermore, the analysis reveals a non-monotonic size sensitivity of the phason field under certain foundation constraints, and the corresponding energy decomposition suggests that this transition is governed by the relative competition between the foundation energy and the couple-stress energy. These observations are not readily obtained by simply combining existing theoretical ingredients and may provide additional useful insight for the mechanical analysis of quasicrystal microbeams on elastic foundations.

## 2. A Theoretical Model of 1D Piezoelectric Quasicrystal Microbeams on a Winkler–Pasternak Foundation

### 2.1. Equations of Motion and Boundary Conditions

**Figure 1:**
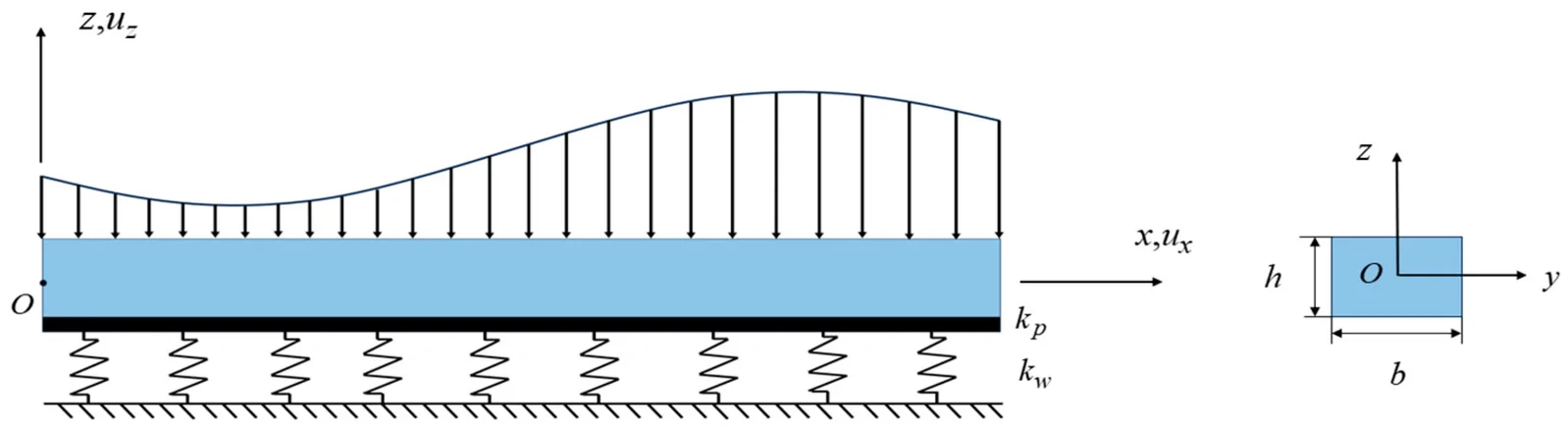
A schematic diagram of a 1D hexagonal piezoelectric quasicrystal microbeam.

A Timoshenko microbeam made of a 1D hexagonal piezoelectric quasicrystal and resting on a Winkler–Pasternak foundation is considered. The beam has length *L*, width *b*, and height *h*. The *z*-axis is aligned with the quasicrystal periodic direction and the poling direction. The Cartesian coordinate system is illustrated in Figure 1. 

Based on the linear elasticity of 1D quasicrystals and the modified couple stress theory [41,47], the geometric relations for the beam are expressed as

(1) $$\begin{array}{l} \varepsilon_{ij}=\frac{1}{2}\left(u_{i,j}+u_{j,i}\right),\;\omega_{3j}=w_{3,j},\\ \chi_{ij}=\frac{1}{2}\left(\theta_{i,j}+\theta_{j,i}\right),\;E_{j}=-\Phi_{,j} \end{array}$$

In these expressions, *u_i_* (*i* = 1, 2, 3) and *w*_3_ denote the elastic displacements associated with the phonon and phason fields, respectively. The quantities *ε_ij_*, *ω*_3*j*_ represent the strain tensors of the phonon and phason fields, while *E_j_* and Φ stand for the electric field components and the electric potential, respectively. *χ_ij_* is the curvature tensor and *θ_i_* denotes the component of the rotation vector [47], defined by

(2) $$\theta_{i}=\frac{1}{2}\varepsilon_{ijk}u_{k,j},$$

We extend the Timoshenko first-order shear deformation beam theory to quasicrystal materials [40,48]. The displacement field and electric potential are expressed as

(3) $$\begin{array}{l} u_{1}=u_{x}\left(x,t\right)-z\phi \left(x,t\right),\;u_{2}=0,\;u_{3}=u_{z}\left(x,t\right),\\ w_{3}=w_{z}\left(x,t\right),\;\Phi =-\cos\left(\frac{\pi }{h}z\right)\gamma \left(x,t\right)+\frac{2z}{h}\gamma_{0} \end{array}$$

where $u_{x}\left(x,t\right)$ and $u_{z}\left(x,t\right)$ denote the axial displacement and deflection of the phonon field at a point on the neutral axis (*y* = 0, *z* = 0) of the beam cross-section at time *t*, respectively; $\phi \left(x,t\right)$ is the rotation angle of the beam cross-section relative to the *z*-axis at the same point; $w_{z}\left(x,t\right)$ represents the transverse displacement of the phason at the same point and time; and $\gamma \left(x,t\right)$ denotes the spatial variation in the electric potential along the *x*-direction at time *t*, while γ_0_ is the externally applied electric potential.

Substituting Equation (3) into Equation (1) yields

(4) $$\begin{array}{l} \varepsilon_{xx}=\frac{\partial u_{x}}{\partial x}-z\frac{\partial \phi }{\partial x},\;\varepsilon_{zx}=\frac{1}{2}\left(\frac{\partial u_{z}}{\partial x}-\phi \right),\;\omega_{zx}=\frac{\partial w_{z}}{\partial x},\\ \chi_{xy}=-\frac{1}{4}\left(\frac{\partial^{2}u_{z}}{\partial x^{2}}+\frac{\partial \phi }{\partial x}\right),\;E_{x}=\cos\left(\frac{\pi }{h}z\right)\frac{\partial \gamma }{\partial x},\;E_{z}=-\frac{\pi }{h}\sin\left(\frac{\pi }{h}z\right)\gamma -\frac{2}{h}\gamma_{0} \end{array}$$

The constitutive relations for this microbeam take the form [47,48,49]:

(5) $$\begin{array}{l} \sigma_{xx}=C_{11}\varepsilon_{xx}-e_{13}E_{z},\\ \sigma_{zx}=2C_{44}\varepsilon_{zx}+R_{3}\omega_{zx}-e_{15}E_{x},\\ H_{zx}=2R_{3}\varepsilon_{zx}+K_{2}\omega_{zx}-d_{15}E_{x},\\ m_{xy}=2Gl^{2}\chi_{xy},\\ D_{x}=2e_{15}\varepsilon_{zx}+d_{15}\omega_{zx}+\lambda_{11}E_{x},\\ D_{z}=e_{13}\varepsilon_{xx}+\lambda_{33}E_{z}, \end{array}$$

In the above equations, $\sigma_{ij}$ and $H_{ij}$ denote the stress tensors of the phonon field and phason field, respectively; $m_{ij}$ is the component of the couple stress tensor; and $D_{i}$ is the electric displacement vector. The quantities $C_{ij},K_{i},R_{i}$ correspond to the elastic constants of the phonon field, the elastic constants of the phason field, and phonon–phason coupling constants; $e_{ij},d_{ij}$ are the piezoelectric constants of the phonon field and the phason field; $\lambda_{ij}$ is the dielectric constant; $G=\left(2C_{44}+C_{66}\right)/3$ is the average shear modulus of the quasicrystal material [47]; and *l* is the material length scale parameter.

In this paper, the Winkler–Pasternak elastic foundation is employed to describe the interaction between the 1D piezoelectric quasicrystal microbeam and the foundation. This two-parameter idealized model [46] consists of continuously distributed normal springs (Winkler springs) together with an incompressible shear layer (the Pasternak layer). The corresponding force–deformation relation is given by

(6) $$F_{w}=k_{w}u_{z},\;T_{p}=k_{p}\frac{\partial u_{z}}{\partial x}$$

where $F_{w}$ and $T_{p}$ represent the Winkler spring force and the shear force of the Pasternak shear layer, respectively; $k_{w}$ is the Winkler spring stiffness, and $k_{p}$ is the Pasternak shear layer stiffness.

Over the interval [0, *T*], the total strain energy, including the modified couple stress contribution and the foundation effect, is expressed as [40,41]

(7) $$\begin{array}{ccc} U & = & \frac{1}{2}\int_{0}^{L}\int_{A}\left(\sigma_{xx}\varepsilon_{xx}+2\sigma_{zx}\varepsilon_{zx}+2m_{xy}\chi_{xy}+H_{zx}\omega_{zx}-D_{x}E_{x}-D_{z}E_{z}\right)dAdx\\ & & +\frac{1}{2}\int_{0}^{L}\left(F_{w}u_{z}+T_{p}u_{z,x}\right)dx \end{array}$$

where *L* and *A* represent the beam length and cross-sectional area, respectively.

Varying the total potential energy of the 1D piezoelectric quasicrystal Timoshenko microbeam over [0, *T*] yields

(8) $$\begin{array}{ccc} \delta \int_{0}^{T}Udt & = & \int_{0}^{T}\int_{0}^{L}\int_{A}\left(\sigma_{xx}\delta \varepsilon_{xx}+2\sigma_{zx}\delta \varepsilon_{zx}+2m_{xy}\delta \chi_{xy}+H_{zx}\delta \omega_{zx}-D_{x}\delta E_{x}-D_{z}\delta E_{z}\right)dAdxdt\\ & & +\int_{0}^{T}\int_{0}^{L}\left(F_{w}\delta u_{z}+T_{p}\delta u_{z,x}\right)dxdt\\ & = & \int_{o}^{T}\int_{0}^{L}\left[-\frac{\partial N_{xx}}{\partial x}\delta u_{x}-\left(\frac{\partial N_{zx}}{\partial x}+\frac{1}{2}\frac{\partial^{2}Y_{xy}}{\partial x^{2}}-k_{w}u_{z}+k_{p}\frac{\partial^{2}u_{z}}{\partial x^{2}}\right)\delta u_{z}\right.\\ & & \left.+\left(\frac{\partial M_{xx}}{\partial x}-N_{zx}+\frac{1}{2}\frac{\partial Y_{xy}}{\partial x}\right)\delta \phi -\left(\frac{\partial T_{zx}}{\partial x}\right)\delta w_{z}+\left(\frac{\partial V_{x}}{\partial x}+V_{z}\right)\delta \gamma \right]dxdt\\ & & +\int_{0}^{T}\left[N_{xx}\delta u_{x}+\left(N_{zx}+\frac{1}{2}\frac{\partial Y_{xy}}{\partial x}+k_{p}\frac{\partial u_{z}}{\partial x}\right)\delta u_{z}-\left(M_{xx}+Y_{xy}\right)\delta \phi \right.\\ & & {\left.+T_{zx}\delta w_{z}-\frac{1}{2}Y_{xy}\frac{\partial \delta u_{z}}{\partial x}-D_{x}\delta \gamma \right]}_{x=0}^{x=L}dt \end{array}$$

in which

(9) $$N_{xx}=\int_{A}\sigma_{xx}dA,\;\;M_{xx}=\int_{A}\sigma_{xx}zdA,\;\;N_{zx}=\int_{A}\sigma_{zx}dA,\;\;T_{zx}=\int_{A}H_{zx}dA,$$

are the phonon-field and phason-field stress resultants on the beam cross-section, respectively, and

(10) $$Y_{xy}=\int_{A}m_{xy}dA,\;\;V_{x}=\int_{A}D_{x}\cos\left(\frac{\pi }{h}z\right)dA,\;\;V_{z}=\int_{A}D_{z}\frac{\pi }{h}\sin\left(\frac{\pi }{h}z\right)dA,$$

are the couple stresses and electric resultants on the beam cross-section, respectively. Specifically,

(11) $$\begin{array}{l} N_{xx}=A\left(C_{11}\frac{\partial u_{x}}{\partial x}+\frac{2}{h}e_{13}\gamma_{0}\right),\;\;M_{xx}=-IC_{11}\frac{\partial \phi }{\partial x}+\frac{2A}{\pi }e_{13}\gamma ,\\ N_{xz}=A\left[k_{s}^{2}C_{44}\left(\frac{\partial u_{z}}{\partial x}-\phi \right)+k_{s}R_{3}\frac{\partial w_{z}}{\partial x}-\frac{2}{\pi }k_{s}e_{15}\frac{\partial \gamma }{\partial x}\right],\\ T_{zx}=A\left[k_{s}R_{3}\left(\frac{\partial u_{z}}{\partial x}-\phi \right)+K_{2}\frac{\partial w_{z}}{\partial x}-\frac{2}{\pi }d_{15}\frac{\partial \gamma }{\partial x}\right], \end{array}$$

(12) $$\begin{array}{l} Y_{xy}=-\frac{Gl^{2}A}{2}\left(\frac{\partial^{2}u_{z}}{\partial x^{2}}+\frac{\partial \phi }{\partial x}\right),\\ V_{x}=A\left[\frac{2k_{s}e_{15}}{\pi }\left(\frac{\partial u_{z}}{\partial x}-\phi \right)+\frac{2}{\pi }d_{15}\frac{\partial w_{z}}{\partial x}+\frac{\lambda_{11}}{2}\frac{\partial \gamma }{\partial x}\right],\\ V_{z}=-A\left(\frac{2e_{13}}{\pi }\frac{\partial \phi }{\partial x}+\frac{\lambda_{33}\pi^{2}}{2h^{2}}\gamma \right), \end{array}$$

where $I=\int_{A}z^{2}dA$ and $k_{s}$ represent the moment of inertia of the beam cross-section and the shear correction factor, respectively; the shear correction factor accounts for the complex shear deformation caused by the non-uniform distribution of shear stress over the cross-section, and is taken as $k_{s}=\sqrt{0.8}$ [50].

Since the phason displacement does not represent a physical displacement of atoms but rather describes the phason degree of freedom that characterizes atomic rearrangement within quasicrystals [2], it differs fundamentally from the phonon field displacements *u_x_* and *u_z_*, which represent macroscopic displacements. Because the phason field possesses no associated inertial mass, it makes no contribution to the kinetic energy. Over the time interval [0, *T*], the kinetic energy of the present beam model [41] takes the form:

(13) $$K=\frac{1}{2}\int_{V}\rho \left[{\left(\frac{\partial u_{1}}{\partial t}\right)}^{2}+{\left(\frac{\partial u_{3}}{\partial t}\right)}^{2}\right]dV$$

where ρ is the mass density of the beam. Its first variation is

(14) $$\begin{array}{c} \delta \int_{0}^{T}Kdt=-\int_{0}^{T}\int_{0}^{L}\int_{A}\rho \left(\frac{\partial^{2}u_{x}}{\partial t^{2}}\delta u_{x}+z^{2}\frac{\partial^{2}\phi }{\partial t^{2}}\delta \phi +\frac{\partial^{2}u_{z}}{\partial t^{2}}\delta u_{z}\right)dAdxdt\\ =-\int_{0}^{T}\int_{0}^{L}\left(m_{0}\frac{\partial^{2}u_{x}}{\partial t^{2}}\delta u_{x}+m_{2}\frac{\partial^{2}\phi }{\partial t^{2}}\delta \phi +m_{0}\frac{\partial^{2}u_{z}}{\partial t^{2}}\delta u_{z}\right) \end{array}$$

with

(15) $$m_{0}=\int_{A}\rho dA,m_{2}=\int_{A}\rho z^{2}dA,$$

As applying phason field loads is currently impractical [17], only the axial load acting on the phonon field is considered in this paper. According to the modified couple stress theory, the virtual work done by the external forces acting on the beam over the time interval [0-*T*] is given by [51]

(16) $$\begin{array}{ccc} \delta \int_{0}^{T}Wdt & = & \int_{0}^{T}\int_{0}^{L}\left(f_{x}\delta u_{x}+f_{z}\delta u_{z}+c\delta \theta_{y}\right)dxdt\\ & & +{\int_{0}^{T}\left.\left(\bar{N_{1}}\delta u_{x}+\bar{N_{2}}\delta u_{z}-\bar{M}\delta \phi \right)\right|}_{x=0}^{x=L}dt\\ & = & \int_{0}^{T}\int_{0}^{L}\left(f_{x}\delta u_{x}+f_{z}\delta u_{z}+\frac{1}{2}\frac{\partial c}{\partial x}\delta u_{z}-\frac{1}{2}c\delta \phi \right)dxdt\\ & & +{\int_{0}^{T}\left.\left(\bar{N_{1}}\delta u_{x}+\bar{N_{2}}\delta u_{z}-\bar{M}\delta \phi -\frac{1}{2}c\delta u_{z}\right)\right|}_{x=0}^{x=L}dt \end{array}$$

where $f_{x}$ and $f_{z}$ are the components of the body force per unit length in the *x*- and *z*-directions, respectively, and *c* denotes the component of the body couple per unit length acting along the *y*-direction. The symbols ${\bar{N}}_{1}$, ${\bar{N}}_{2}$ and $\bar{M}$ rrepresent the axial force, the transverse shear force, and the bending moment applied at the two ends of the beam, respectively.

According to Hamilton’s principle [51,52], the following can be obtained:

(17) $$\delta \int_{0}^{T}\left[K-\left(U-W\right)\right]dt=0$$

Substituting Equations (8), (14) and (16) into Equation (17), application of the fundamental lemma of the calculus of variations, together with the arbitrariness of $\delta u_{x}$, $\delta u_{z}$,δϕ, $\delta w_{z}$ and δγ over the time interval [0, *T*], leads to the following governing equations of motion:

(18) $$\begin{array}{l} \frac{\partial N_{xx}}{\partial x}+f_{x}=m_{0}\frac{\partial^{2}u_{x}}{\partial t^{2}},\\ \frac{\partial N_{zx}}{\partial x}+\frac{1}{2}\frac{\partial^{2}Y_{xy}}{\partial x^{2}}-k_{w}u_{z}+k_{p}\frac{\partial^{2}u_{z}}{\partial x^{2}}+f_{z}+\frac{1}{2}\frac{\partial c}{\partial x}=m_{0}\frac{\partial^{2}u_{z}}{\partial t^{2}},\\ N_{zx}-\frac{\partial M_{xx}}{\partial x}-\frac{1}{2}\frac{\partial Y_{xy}}{\partial x}-\frac{1}{2}c=m_{2}\frac{\partial^{2}\phi }{\partial t^{2}},\\ \frac{\partial T_{zx}}{\partial x}=0,\\ \frac{\partial V_{x}}{\partial x}+V_{z}=0 \end{array}$$

For the present 1D hexagonal piezoelectric quasicrystal Timoshenko beam supported by a Winkler–Pasternak elastic foundation, the boundary conditions are given by

(19) $$\begin{array}{l} N_{xx}=0\;\mathrm{or}\;u_{x}=\bar{u_{x}}\;\mathrm{at}\;x=0\;\mathrm{and}\;x=L,\\ N_{zx}+\frac{1}{2}\frac{\partial Y_{xy}}{\partial x}+k_{p}\frac{\partial u_{z}}{\partial x}=0\;\mathrm{or}\;u_{z}=\bar{u_{z}}\mathrm{at}\;x=0\;\mathrm{and}\;x=L,\\ M_{xx}+\frac{1}{2}Y_{xy}=0\;\mathrm{or}\;\phi =\bar{\phi }\;\mathrm{at}\;x=0\;\mathrm{and}\;x=L,\\ T_{zx}=0\;\mathrm{or}\;w_{z}=\bar{w_{z}}\;\mathrm{at}\;x=0\;\mathrm{and}\;x=L,\\ Y_{xy}=0\;\mathrm{or}\;\frac{\partial u_{z}}{\partial x}=\frac{\bar{\partial u_{z}}}{\partial x}\;\mathrm{at}\;x=0\;\mathrm{and}\;x=L,\\ D_{x}=0\;\mathrm{or}\;\gamma =\bar{\gamma }\;\mathrm{at}\;x=0\;\mathrm{and}\;x=L \end{array}$$

Here, the overline denotes a prescribed constant value. For a specific problem, the required boundary conditions are obtained from the general ones given above.

In this study, the beam is assumed to be electrically open-circuited, with no external electric field and no free charges applied, so that the net electric displacement over the beam thickness vanishes; the electric potential Φ is therefore generated solely by mechanical deformation through the piezoelectric coupling. This open-circuit potential is a genuine piezoelectric output and can be measured experimentally by depositing thin electrodes on the beam surfaces and using a high-impedance voltmeter under high-frequency or electrically insulated configurations. For conductive quasicrystals, this open-circuit potential represents the intrinsic piezoelectric response before charge compensation, and it is physically meaningful for high-frequency or electrically insulated configurations. Consequently, all external electric field terms are omitted from the governing equations of motion. Substituting Equations (11) and (12) into Equation (18), the governing equations of motion for the 1D hexagonal piezoelectric quasicrystal Timoshenko beam resting on the Winkler–Pasternak elastic foundation take the following form:

(20) $$\begin{array}{l} AC_{11}\frac{\partial^{2}u_{x}}{\partial x^{2}}+f_{x}=m_{0}\frac{\partial^{2}u_{x}}{\partial t^{2}},\\ Ak_{s}^{2}C_{44}\left(\frac{\partial^{2}u_{z}}{\partial x^{2}}-\frac{\partial \phi }{\partial x}\right)-\frac{Gl^{2}A}{4}\left(\frac{\partial^{4}u_{z}}{\partial x^{4}}+\frac{\partial^{3}\phi }{\partial x^{3}}\right)+Ak_{s}R_{3}\frac{\partial^{2}w_{z}}{\partial x^{2}}-\frac{2A}{\pi }k_{s}e_{15}\frac{\partial^{2}\gamma }{\partial x^{2}}\\ -k_{w}u_{z}+k_{p}\frac{\partial^{2}u_{z}}{\partial x^{2}}+f_{z}+\frac{1}{2}\frac{\partial c}{\partial x}=m_{0}\frac{\partial^{2}u_{z}}{\partial t^{2}},\\ Ak_{s}^{2}C_{44}\left(\frac{\partial u_{z}}{\partial x}-\phi \right)+IC_{11}\frac{\partial^{2}\phi }{\partial x^{2}}+\frac{Gl^{2}A}{4}\left(\frac{\partial^{3}u_{z}}{\partial x^{3}}+\frac{\partial^{2}\phi }{\partial x^{2}}\right)+Ak_{s}R_{3}\frac{\partial w_{z}}{\partial x}\\ -\frac{2A}{\pi }k_{s}e_{15}\frac{\partial \gamma }{\partial x}-\frac{2A}{\pi }e_{13}\frac{\partial \gamma }{\partial x}-\frac{1}{2}c=m_{2}\frac{\partial^{2}\phi }{\partial t^{2}},\\ Ak_{s}R_{3}\left(\frac{\partial^{2}u_{z}}{\partial x^{2}}-\frac{\partial \phi }{\partial x}\right)+AK_{2}\frac{\partial^{2}w_{z}}{\partial x^{2}}-\frac{2A}{\pi }d_{15}\frac{\partial^{2}\gamma }{\partial x^{2}}=0,\\ -\frac{2A}{\pi }k_{s}e_{15}\left(\frac{\partial^{2}u_{z}}{\partial x^{2}}-\frac{\partial \phi }{\partial x}\right)+\frac{2A}{\pi }e_{13}\frac{\partial \phi }{\partial x}-\frac{2A}{\pi }d_{15}\frac{\partial^{2}w_{z}}{\partial x^{2}}\\ -\frac{A\lambda_{11}}{2}\frac{\partial^{2}\gamma }{\partial x^{2}}+\frac{A\lambda_{33}\pi^{2}}{2h^{2}}\gamma =0, \end{array}$$

For any *x* ∈ [0, *L*] and *t* ∈ [0, *T*], it should be noted that $u_{x}\left(x,t\right)$, $u_{z}\left(x,t\right)$, $\phi \left(x,t\right)$, $w_{z}\left(x,t\right)$ and $\gamma \left(x,t\right)$ are obtained by solving the governing equations of motion, Equation (20), together with the boundary conditions given in Equation (19). The governing equations contain the material length scale parameter *l*; therefore, the established quasicrystal Timoshenko beam model inherently incorporates the size effect governed by couple stresses.

### 2.2. Static Bending Analysis

In the static bending problem, the displacements, rotations, and electric potential of the beam are independent of time *t*. The time-dependent terms in the governing equations of motion Equation (20) are therefore neglected. To solve the governing equations, the following Fourier solutions are assumed for $u_{x}\left(x,t\right)$, $u_{z}\left(x,t\right)$, $\phi \left(x,t\right)$, $w_{z}\left(x,t\right)$ and $\gamma \left(x,t\right)$:

(21) $$\begin{array}{l} u_{x}\left(x\right)=\sum_{n=1}^{\infty }a_{0}\cos\left(\frac{n\pi x}{L}\right),\\ u_{z}\left(x\right)=\sum_{n=1}^{\infty }a_{1}\sin\left(\frac{n\pi x}{L}\right),\\ \phi \left(x\right)=\sum_{n=1}^{\infty }a_{2}\cos\left(\frac{n\pi x}{L}\right),\\ w_{z}\left(x\right)=\sum_{n=1}^{\infty }a_{3}\sin\left(\frac{n\pi x}{L}\right),\\ \gamma \left(x\right)=\sum_{n=1}^{\infty }a_{4}\sin\left(\frac{n\pi x}{L}\right), \end{array}$$

In the present example, the body force component $f_{x}$ is prescribed to be zero, and the uniformly distributed load $f_{z}$ can also be assumed as the following Fourier series:

(22) $$f_{z}\left(x\right)=\sum_{n=1}^{\infty }Q_{n}\sin\left(\frac{n\pi x}{L}\right),$$

where $Q_{n}$ is the Fourier coefficient. For the case of a uniformly distributed load $f_{z}\left(x\right)=p_{0}$, we obtain

(23) $$Q_{n}=\frac{2p_{0}}{n\pi }\left[1-\cos\left(n\pi \right)\right],$$

Substituting Equation (21) into Equation (20) yields

(24) $$\left[\begin{array}{ccccc} D_{11} & D_{12} & D_{13} & D_{14} & D_{15}\\ D_{21} & D_{22} & D_{23} & D_{24} & D_{25}\\ D_{31} & D_{32} & D_{33} & D_{34} & D_{35}\\ D_{41} & D_{42} & D_{43} & D_{44} & D_{45}\\ D_{51} & D_{52} & D_{53} & D_{54} & D_{55} \end{array}\right]\left[\begin{array}{c} a_{0}\\ a_{1}\\ a_{2}\\ a_{3}\\ a_{4} \end{array}\right]=\left[\begin{array}{c} 0\\ -Q_{n}\\ 0\\ 0\\ 0 \end{array}\right],$$

where

(25) $$\begin{array}{l} D_{11}=-AC_{11}{\left(\frac{n\pi }{L}\right)}^{2},\;D_{22}=-Ak_{s}^{2}C_{44}{\left(\frac{n\pi }{L}\right)}^{2}-\frac{Gl^{2}A}{4}{\left(\frac{n\pi }{L}\right)}^{4}-k_{p}{\left(\frac{n\pi }{L}\right)}^{2}-k_{w},\\ D_{33}=-\left[IC_{11}+\frac{Gl^{2}A}{4}\right]{\left(\frac{n\pi }{L}\right)}^{2}-Ak_{s}^{2}C_{44},\;D_{44}=-AK_{2}{\left(\frac{n\pi }{L}\right)}^{2},\;D_{45}=\frac{2A}{\pi }d_{15}{\left(\frac{n\pi }{L}\right)}^{2},\\ D_{55}=\frac{A\lambda_{11}}{2}{\left(\frac{n\pi }{L}\right)}^{2}+\frac{A\lambda_{33}\pi^{2}}{2h^{2}},\;D_{12}=D_{21}=D_{13}=D_{31}=D_{14}=D_{41}=D_{15}=D_{51}=0,\\ D_{23}=D_{32}=Ak_{s}^{2}C_{44}\left(\frac{n\pi }{L}\right)-\frac{Gl^{2}A}{4}{\left(\frac{n\pi }{L}\right)}^{3},\;D_{24}=D_{42}=-Ak_{s}R_{3}{\left(\frac{n\pi }{L}\right)}^{2},\\ D_{25}=D_{52}=\frac{2A}{\pi }k_{s}e_{15}{\left(\frac{n\pi }{L}\right)}^{2},\;D_{35}=D_{53}=-2A\left(k_{s}e_{15}+e_{31}\right)\frac{n}{L},\\ D_{34}=D_{43}=Ak_{s}R_{3}\left(\frac{n\pi }{L}\right),\;D_{45}=D_{54}=\frac{2A}{\pi }d_{15}{\left(\frac{n\pi }{L}\right)}^{2}, \end{array}$$

By solving the linear algebraic system in Equation (24), the Fourier coefficients $a_{0}$, $a_{1}$, $a_{2}$, $a_{3}$, $a_{4}$ can be determined. Substituting these Fourier coefficients back into Equation (21), the displacements, rotations, and electric potential of the simply supported beam model can be determined.

### 2.3. Free Vibration Analysis

In the free vibration analysis, all external forces are neglected ($f_{x}=f_{z}=0$, c=0), Consequently, the forcing terms in the governing Equation (20) are omitted. Since $f_{x}=0$, it can be obtained that for any $x\in \left(0,L\right)$, $t\in \left(0,T\right)$ holds in $u_{x}(x,t)=0$, which implies that there is no axial vibration. For $u_{z}\left(x,t\right)$, $\phi \left(x,t\right)$, $w_{z}\left(x,t\right)$ and $\gamma \left(x,t\right)$, the following Fourier solutions are considered:

(26) $$\begin{array}{l} u_{z}\left(x,t\right)=\sum_{n=1}^{\infty }a_{1}^{V}\sin\left(\frac{n\pi x}{L}\right)e^{i\omega_{n}t},\\ \phi \left(x,t\right)=\sum_{n=1}^{\infty }a_{2}^{V}\cos\left(\frac{n\pi x}{L}\right)e^{i\omega_{n}t},\\ w_{z}\left(x,t\right)=\sum_{n=1}^{\infty }a_{3}^{V}\sin\left(\frac{n\pi x}{L}\right)e^{i\omega_{n}t},\\ \gamma \left(x,t\right)=\sum_{n=1}^{\infty }a_{4}^{V}\sin\left(\frac{n\pi x}{L}\right)e^{i\omega_{n}t}, \end{array}$$

Here, $\omega_{n}$ denotes the *n*-th natural frequency, while $a_{1}^{V}$, $a_{2}^{V}$, $a_{3}^{V}$ and $a_{4}^{V}$ are the corresponding Fourier coefficients, and *i* is the imaginary unit. The expansions in Equation (26) satisfy the boundary conditions given in Equation (19) for all t ∈ [0, T]. Substituting Equation (26) into Equation (20) yields

(27) $$\left[\begin{array}{cccc} D_{22} & D_{23} & D_{24} & D_{25}\\ D_{32} & D_{33} & D_{34} & D_{35}\\ D_{42} & D_{43} & D_{44} & D_{45}\\ D_{52} & D_{53} & D_{54} & D_{55} \end{array}\right]\left[\begin{array}{c} a_{1}^{v}\\ a_{2}^{v}\\ a_{3}^{v}\\ a_{4}^{v} \end{array}\right]+\left[\begin{array}{cccc} m_{0}\omega_{n}^{2} & 0 & 0 & 0\\ 0 & m_{2}\omega_{n}^{2} & 0 & 0\\ 0 & 0 & 0 & 0\\ 0 & 0 & 0 & 0 \end{array}\right]\left[\begin{array}{c} a_{1}^{v}\\ a_{2}^{v}\\ a_{3}^{v}\\ a_{4}^{v} \end{array}\right]=\left[\begin{array}{c} 0\\ 0\\ 0\\ 0 \end{array}\right],$$

Since all Fourier coefficients are nonzero, the following determinant is obtained, where the coefficient matrix is the same as that in Equation (25).

(28) $$\left|\begin{array}{cccc} D_{22}+m_{0}\omega_{n}^{2} & D_{23} & D_{24} & D_{25}\\ D_{32} & D_{33}+m_{2}\omega_{n}^{2} & D_{34} & D_{35}\\ D_{42} & D_{43} & D_{44} & D_{45}\\ D_{52} & D_{53} & D_{54} & D_{55} \end{array}\right|=0,$$

By solving the smallest positive root $\omega_{n}^{2}$ of determinant in Equation (28), the *n*-th natural frequency of the present 1D hexagonal piezoelectric quasicrystal Timoshenko microbeam can be obtained.

## 3. Numerical Examples and Results Analysis

### 3.1. Model Validation

To validate the proposed piezoelectric quasicrystal beam model with foundation and its associated solution method, the degenerated results of the present model are compared with the two-dimensional elasticity solutions for simply supported isotropic beams obtained by Chen et al. [53,54] using the state-space integration method. The comparisons are listed in Table 1. The dimensionless natural frequency and foundation stiffness parameters are defined as follows:

(29) $$\sqrt{\bar{\omega_{n}}}=\sqrt{\omega_{n}}{\left(\frac{\rho AL^{4}}{EI}\right)}^{\frac{1}{4}},\;{\bar{k}}_{w}=\frac{k_{w}L^{4}}{EI},\;{\bar{k}}_{p}=\frac{k_{p}L^{2}}{EI},$$

The first natural frequencies obtained from the present model are in excellent agreement with the reference solutions reported by Chen et al. [54], confirming the accuracy of the present formulation in the limiting isotropic beam case.

To further validate the present piezoelectric quasicrystal formulation, the model is degenerated to a simply supported one-dimensional hexagonal piezoelectric quasicrystal Timoshenko microbeam without the elastic foundation by setting $k_{w}=k_{p}=0$. The geometrical and material parameters are taken to be the same as those used by Li and Xiao [40], and a zero external voltage *V* = 0 is considered. Table 2 compares the first three natural frequencies obtained from the present model with the results reported by Li and Xiao [40] for two span-to-height ratios, *L*/*h* = 20 and 30, under different values of the material length scale parameter.

The good agreement shown in Table 2 confirms that the present formulation correctly captures the size-dependent free vibration behavior of piezoelectric quasicrystal microbeams, including the first three natural frequencies.

### 3.2. Static Bending Response

**Figure 2:**
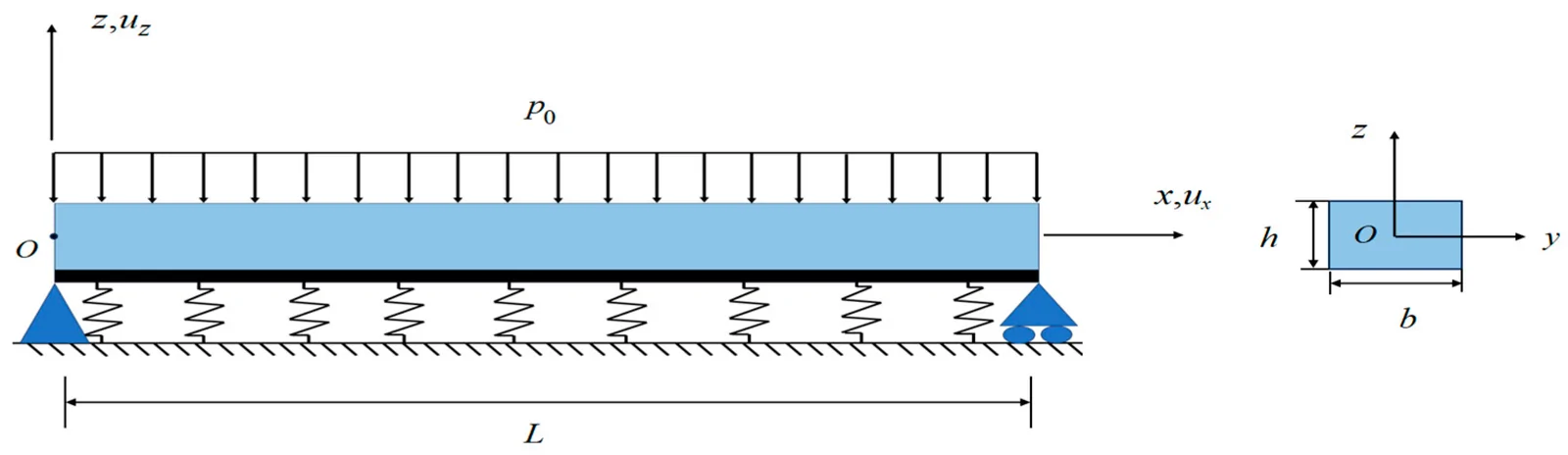
A schematic diagram of a simply supported 1D hexagonal piezoelectric quasicrystal microbeam subjected to a uniformly distributed load.

To investigate the static bending response of the 1D hexagonal piezoelectric quasicrystal Timoshenko microbeam, a simply supported beam with a rectangular cross-section subjected to a uniformly distributed load, as shown in Figure 2, is considered in this section.

According to Equation (19), the boundary conditions for the simply supported microbeam are given by

(30) $$u_{x}=u_{z}=T_{zx}=M_{xx}=Y_{xy}=\gamma =0\;\;\mathrm{at}\;\;x=0\;\;\mathrm{or}\;\;x=L$$

The microbeam considered in this example is made of Al-Ni-Co quasicrystal (QC1) [17,55], with material parameters listed in Table 3. QC1 exhibits transverse isotropy, with the *z*-axis serving as the symmetry axis. The beam thickness is taken as h=10 μm, the width as *b* = 2*h*, and the length as *L* = 20*h*. A uniformly distributed load of 1N/m is applied. Since accurate experimental data for the material length scale parameter *l* of QC1 are currently unavailable, values of *l* = 0, 1.5, 3, and 4.5 μm (corresponding to *l*/*h* = 0, 0.15, 0.3, and 0.45) are adopted in the present study. This range is consistent with existing investigations on size-dependent quasicrystal structures (e.g., [17,24]), where *l*/*h* ratios up to 0.5 have been commonly assumed. The chosen values therefore fall within the physically reasonable interval and allow a systematic examination of the size effect. The foundation stiffness is nondimensionalized as ${\bar{k}}_{w}=80$ and ${\bar{k}}_{p}=20$, with the dimensionless values taken as ${\bar{k}}_{w}=k_{w}L^{4}/\left(C_{11}I\right)$ and ${\bar{k}}_{p}=k_{p}L^{2}/\left(C_{11}I\right)$. When *l* = 0 and $k_{w}=k_{p}=0$, both the size effect and foundation effect vanish in the present beam model, and the beam reduces to the classical model.

The units of $C_{ij}$, $K_{i}$ and $R_{i}$ are $10^{9}N/m^{2}$, those of $e_{ij}$ and $d_{ij}$ are $C/m^{2}$, the unit of $\lambda_{ij}$ is $10^{-9}C^{2}/\left(N\cdot m^{2}\right)$, and the unit of ρ is $10^{3}\mathrm{kg}/m^{2}$.

**Figure 3:**
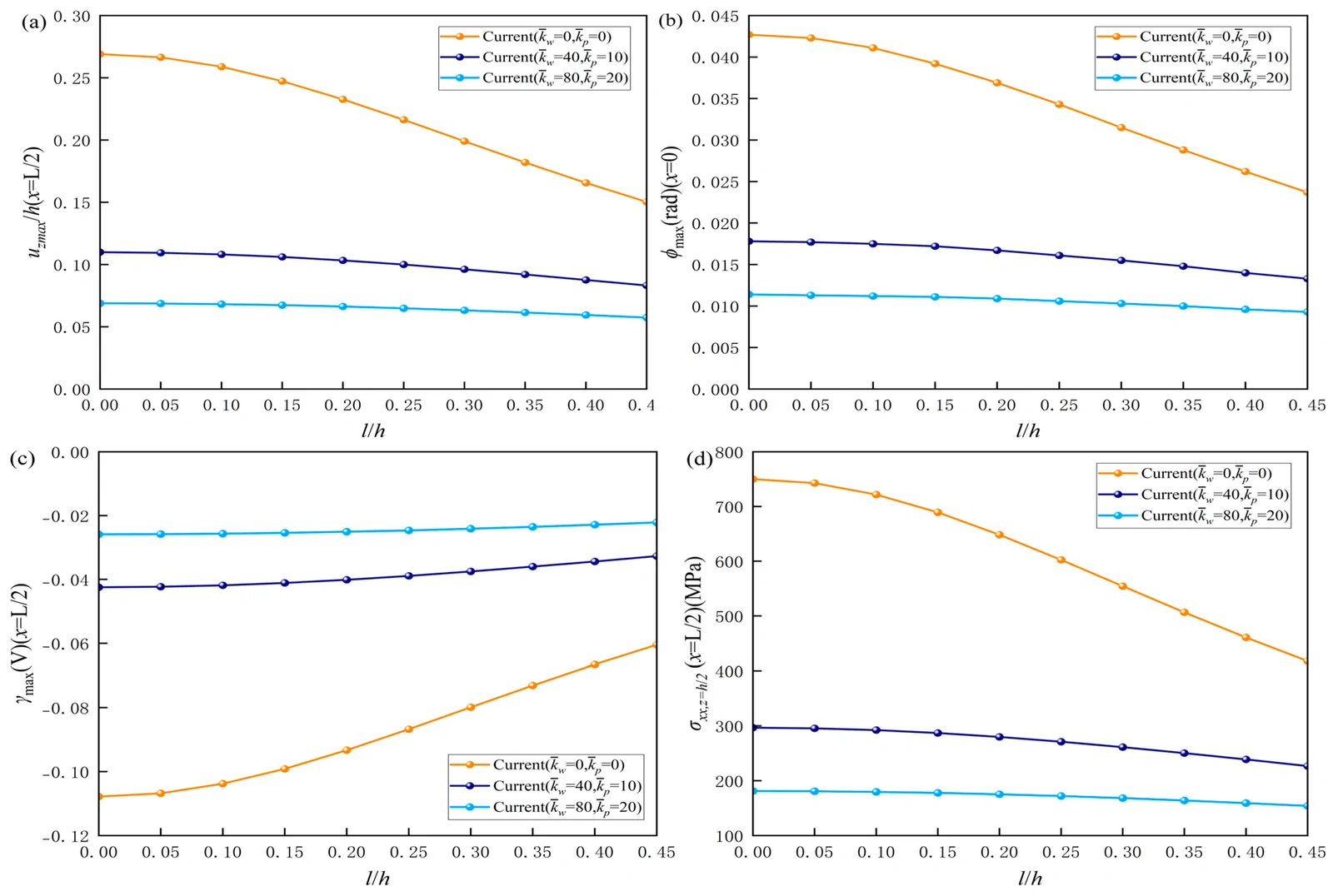
Sensitivity analysis of maximum static responses with respect to *l*/*h* under different foundation parameter combinations: (**a**) maximum phonon displacement; (**b**) maximum phonon-field rotation; (**c**) maximum phonon stress; (**d**) maximum electric potential. Three representative cases are considered: (${\bar{k}}_{w},{\bar{k}}_{p}$) = (0,0), (40,10), and (80,20).

Before presenting the detailed distribution curves, a sensitivity analysis is first conducted to verify the appropriateness of the selected *l*/*h* range. Figure 3 shows the maximum values of the phonon displacement, phonon-field rotation, phonon stress, and electric potential as functions of *l*/*h* for three representative foundation parameter combinations: (${\bar{k}}_{w},{\bar{k}}_{p}$) = (0,0), (40,10), and (80,20).

It is seen that all these quantities vary smoothly and monotonically with *l*/*h* over the entire investigated interval, and no abrupt changes or singularities are observed. This monotonic behavior indicates that the chosen range 0≤l/h≤0.45 is physically reasonable, and that the static predictions are not unduly sensitive to the exact value of the material length scale parameter. The subsequent results are therefore presented for the selected discrete values of *l*/*h* within this range.

**Figure 4:**
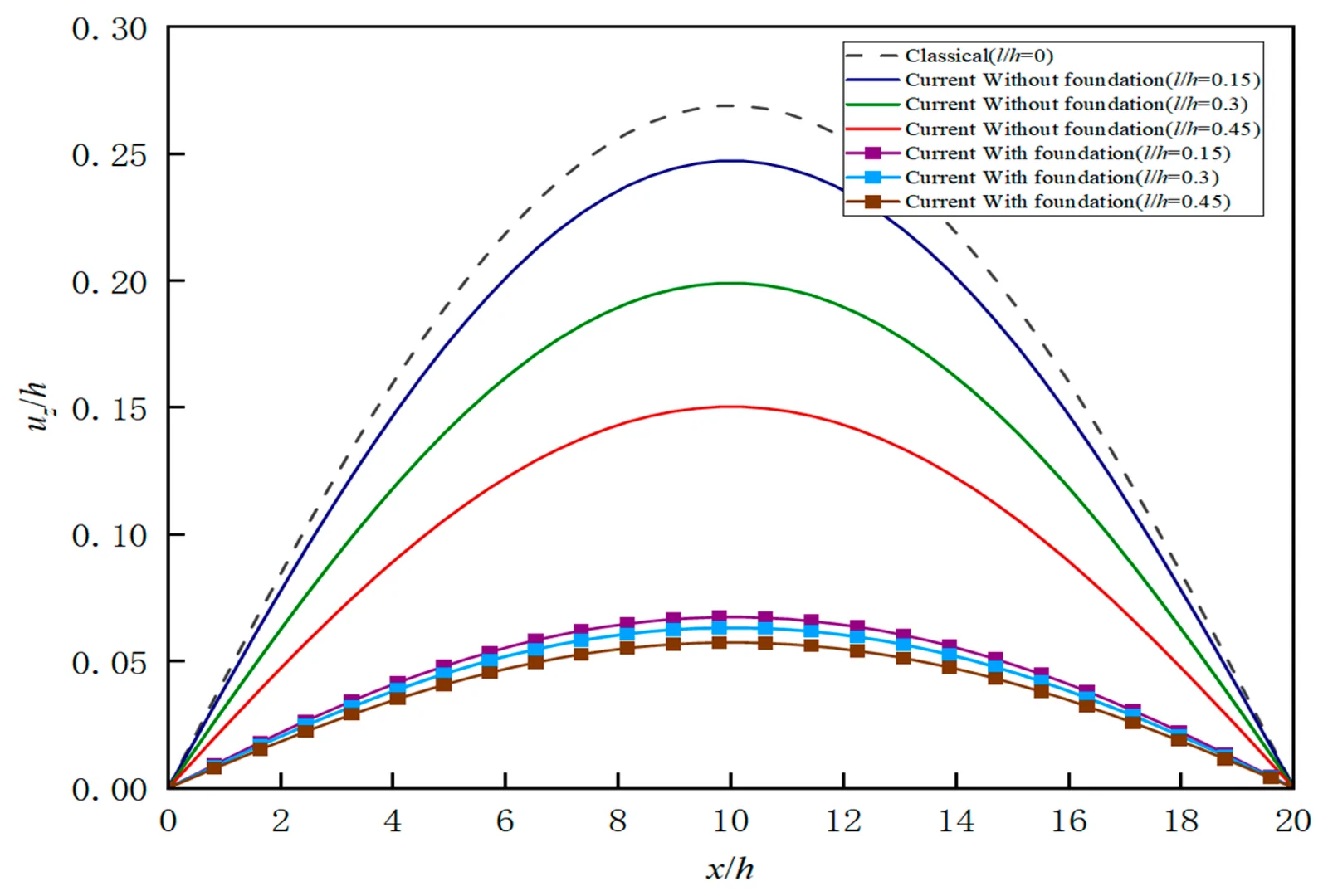
Phonon displacement of 1D hexagonal piezoelectric quasicrystal microbeams for different material length scale parameters *l*/*h.*

**Figure 5:**
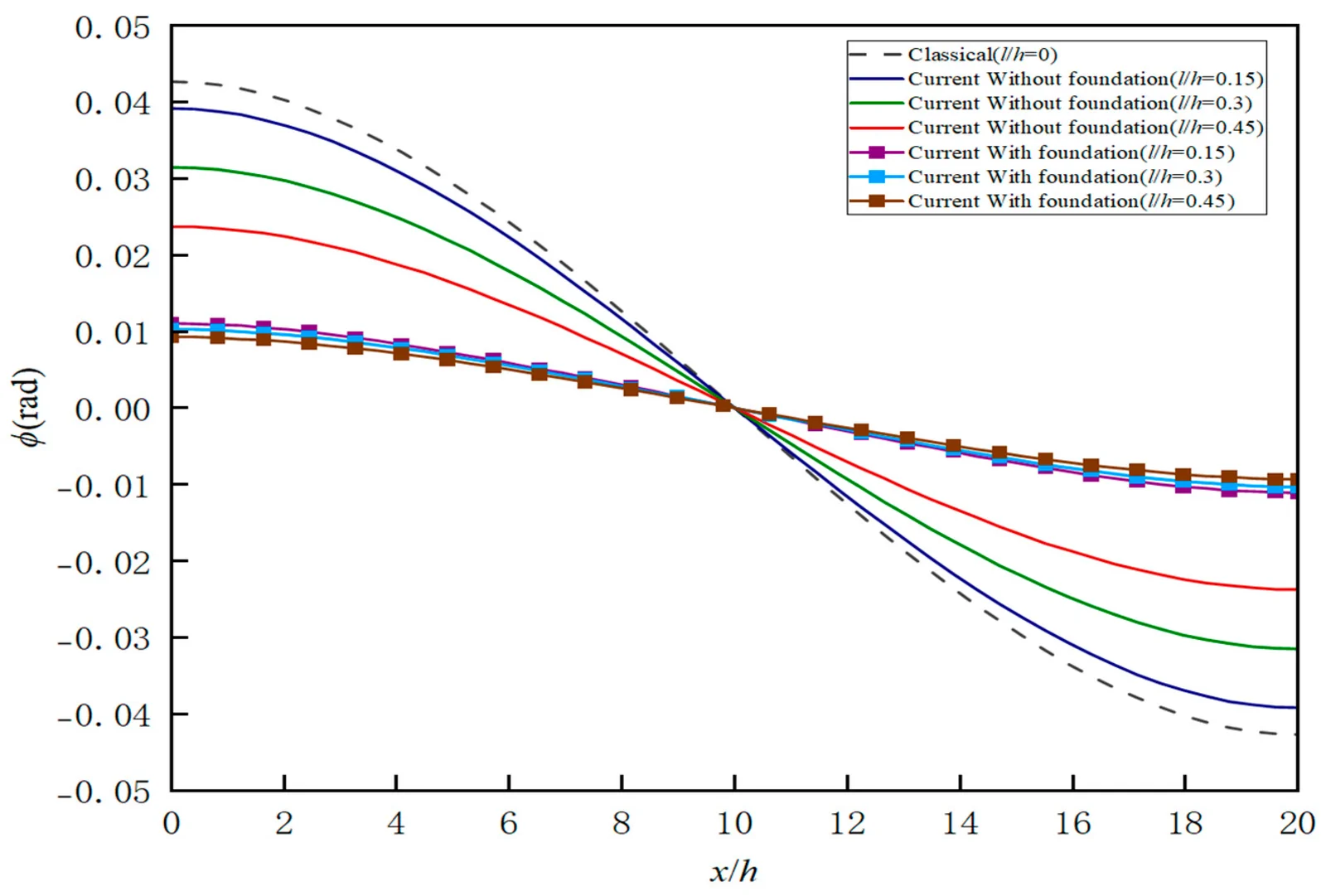
Phonon-field rotation of 1D hexagonal piezoelectric quasicrystal microbeams for different material length scale parameters *l*/*h.*

**Figure 6:**
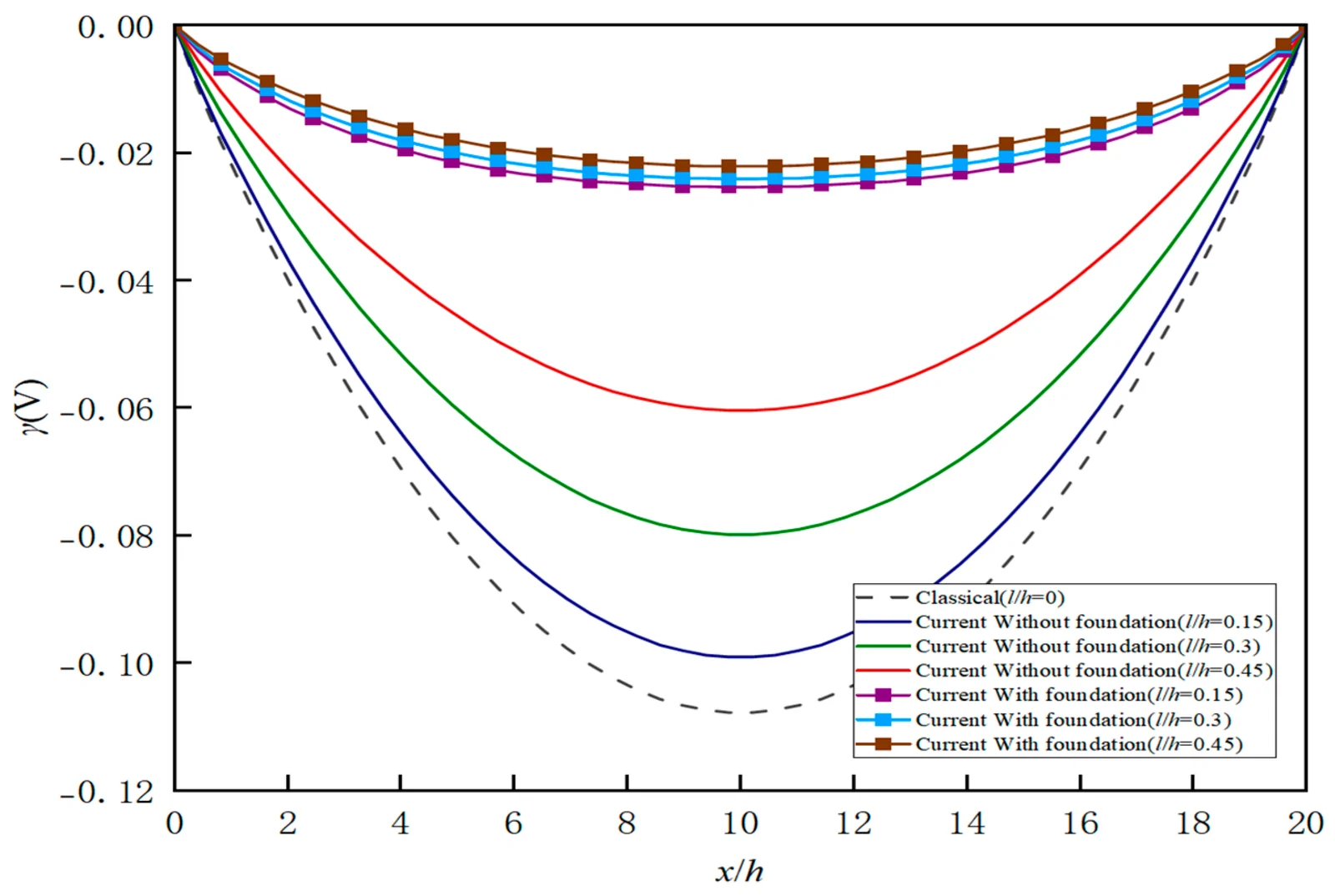
Electric potential of 1D hexagonal piezoelectric quasicrystal microbeams for different material length scale parameters *l*/*h.*

**Figure 7:**
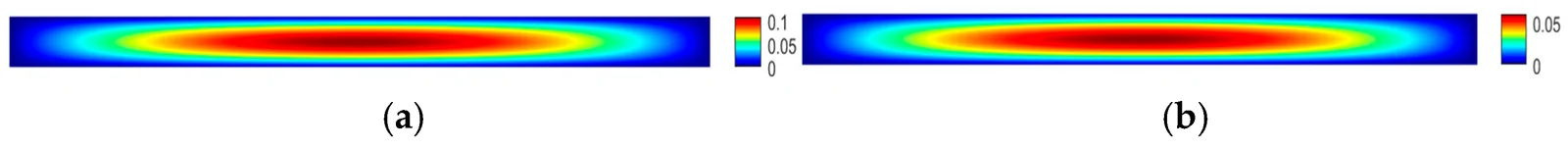
Two-dimensional contour plots of the electric potential Φ on the x-z cross-section of simply supported 1D hexagonal piezoelectric quasicrystal beams with different length scale parameters without a foundation effect: (**a**) *l*/*h* = 0; (**b**) *l*/*h* = 0.45.

**Figure 8:**
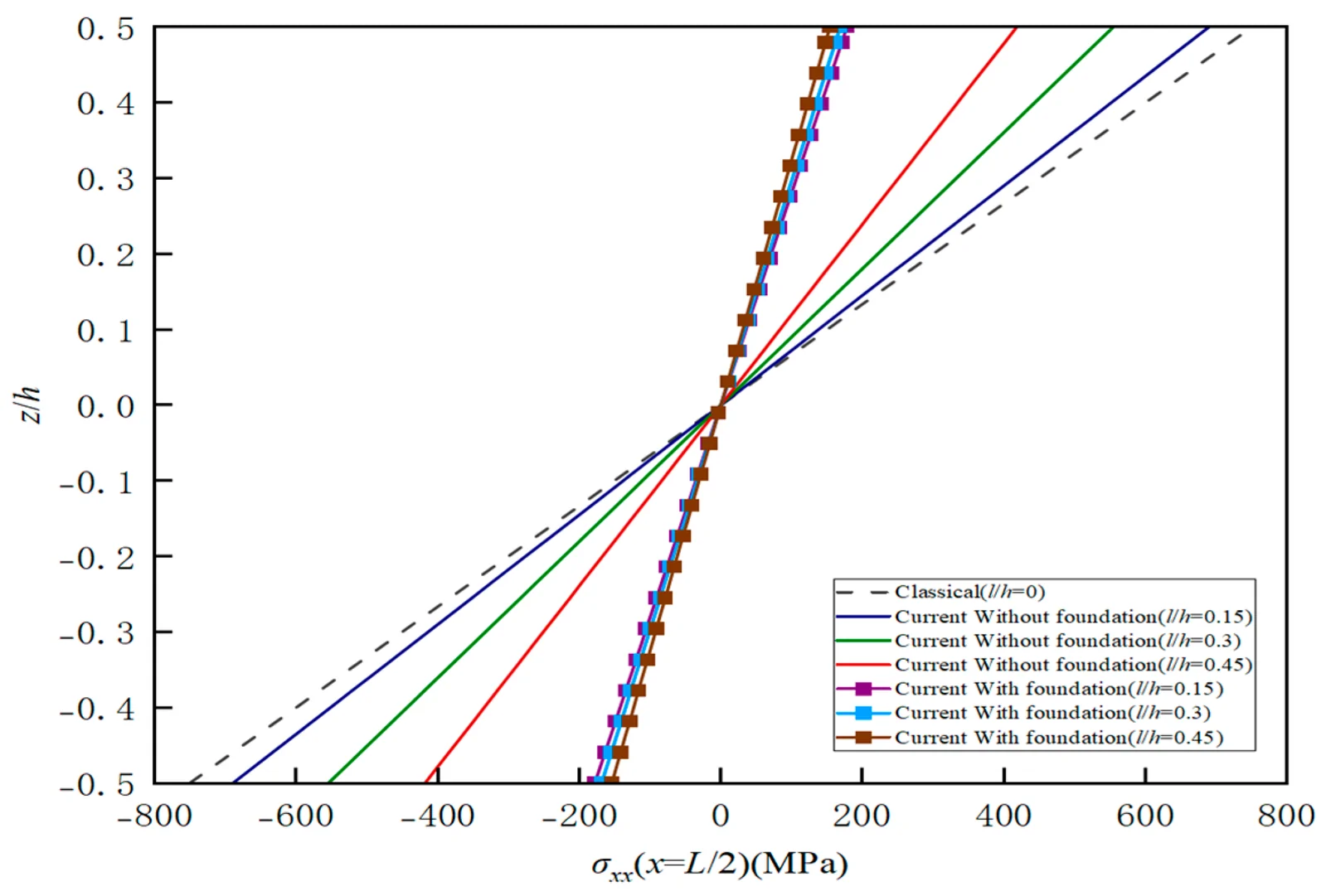
Phonon stress *σ_xx_* at the beam center (*x* = *L*/2) of 1D hexagonal piezoelectric quasicrystal microbeams for different material length scale parameters *l*/*h.*

**Figure 9:**
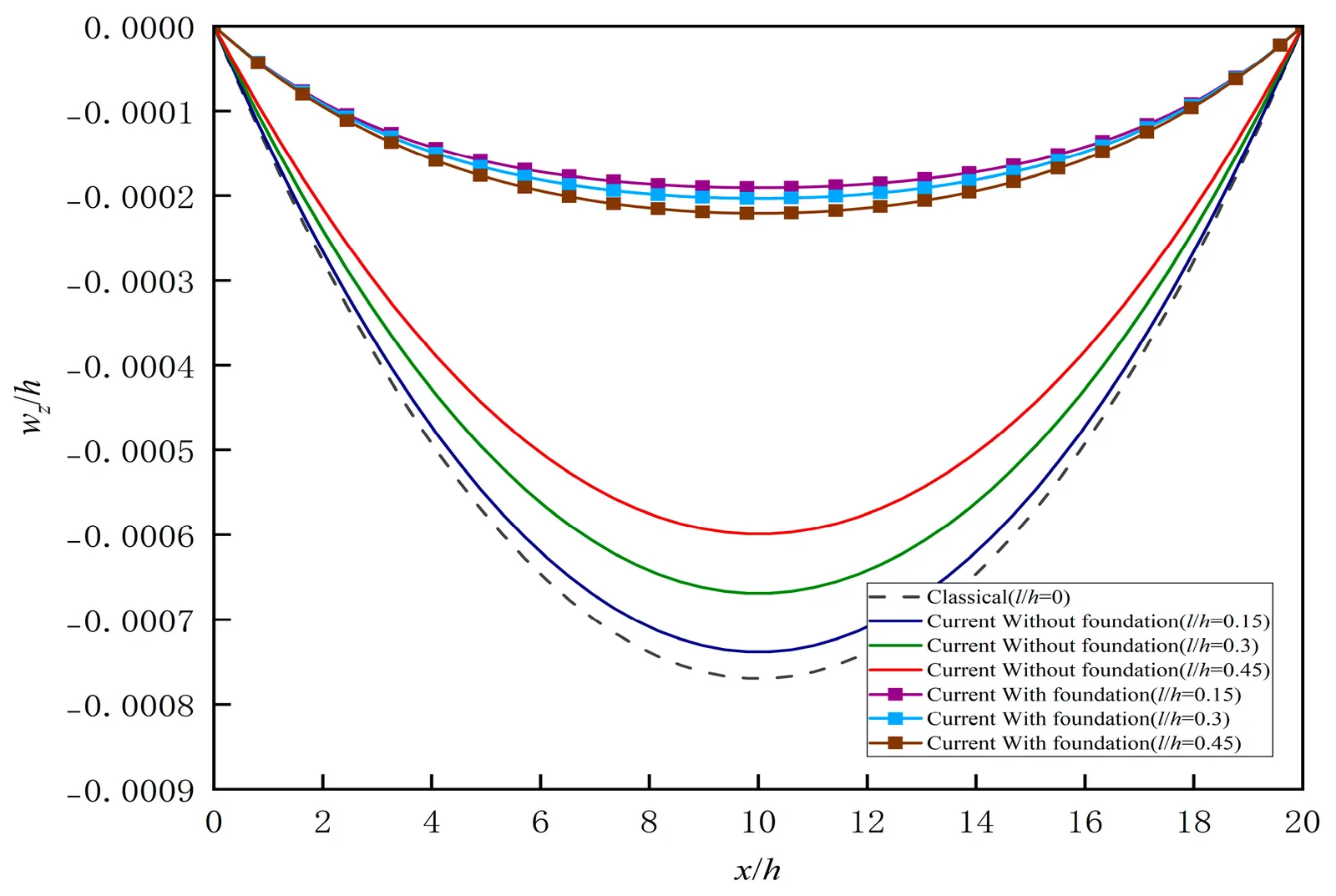
Phason displacement of 1D hexagonal piezoelectric quasicrystal microbeams for different material length scale parameters *l*/*h.*

Figure 4, Figure 5, Figure 6, Figure 7, Figure 8 and Figure 9 present the predicted phonon displacement, phonon-field rotation, electric potential, axial normal stress, and phason displacement for a simply supported 1D hexagonal piezoelectric quasicrystal beam under three models: the classical beam model, the beam model incorporating the size effect, and the beam model incorporating both the size effect and the Winkler–Pasternak foundation effect.

As can be seen from Figure 4 and Figure 5, compared with the classical beam model, the absolute values of the phonon displacement and phonon-field rotation are substantially reduced when the size effect is taken into account, and they continue to decrease as *l*/*h* (i.e., the size effect) increases. After the Winkler–Pasternak elastic foundation is introduced, both the phonon displacement and the phonon-field rotation are further suppressed, directly reflecting the additional constraint exerted by the foundation. 

Figure 6 presents the electric potential curves for the three beam models at various values of the material length scale parameter. It shows that the electric potential predicted by the present non-classical beam model, both with and without the foundation, remains consistently lower than that obtained from the classical model. The electric potential decreases as *l*/*h* (i.e., the size effect) increases and is further reduced by the introduction of the foundation. This indicates that the foundation not only suppresses the mechanical deformation of the beam but also weakens the piezoelectric response through stiffness coupling. It should be emphasized that the electric potential shown in Figure 6 is not an imposed quantity but an intrinsic consequence of the phonon deformation. In the absence of an external electric field, the phonon strains generate an electric field through the piezoelectric constants $e_{31}$ and $e_{15}$, and the resulting spatial variation in Φ represents the open-circuit piezoelectric output of the beam. From an experimental viewpoint, this potential distribution can be measured by fabricating segmented electrodes on the top and bottom surfaces or by using scanning probe techniques. Since the open-circuit condition corresponds to a high-impedance measurement, the obtained Φ is directly related to the voltage that would be recorded by a voltmeter in the absence of significant charge leakage. Therefore, the values reported in this paper are not merely mathematical artifacts; they represent a measurable piezoelectric response.

In Figure 7, the electric potential exhibits a centrally symmetric distribution within the beam cross-section, with the maximum absolute value appearing in the central region and approaching zero at the boundaries. Whether or not the size effect is considered, the maximum absolute value of the electric potential is always located near the geometric center of the beam (*x* = *L*/2, *z* = 0) and decays gradually outward. A comparison of Figure 7a,b reveals that the size effect noticeably changes the amplitude of the electric potential while its spatial distribution pattern remains unchanged. These results indicate that the size effect governed by couple stress suppresses the piezoelectric output without affecting the distribution characteristics of the electric potential field.

Figure 8 shows the distribution of the phonon stress *σ_xx_* along the thickness at the beam center (*x* = *L*/2) for the three beam models under various values of the material length scale parameter. At the central cross-section, the size effect reduces the peak phonon stresses on the top and bottom surfaces. When the foundation is introduced, the phonon stress distribution becomes more uniform, and the surface stress peaks decrease further. This suggests that the foundation constraint indirectly influences the phonon stress distribution by modifying the overall deformation of the beam.

Figure 9 displays the phason displacement curves for the three beam models at different material length scale parameters. The figure reveals a unique response of the phason field. In the absence of a foundation, the phason displacement tends to decrease as the material length scale parameter *l* (or *l*/*h*) increases; however, once the foundation is introduced, the phason displacement tends to stabilize or even rise with increasing *l*/*h*, contrasting sharply with the monotonic variation observed in the phonon displacement. As noted by Liu et al. [53] in their study on bilinear segmented systems with nonlinear constraints, the introduction of constraints serves as an important mechanism for inducing non-classical and non-monotonic responses in a system. The phonon field governs macroscopic elastic deformation, and its size-induced stiffening (phonon displacement decreasing with increasing material length scale parameter) follows classical behavior. In contrast, the phason field describes atomic rearrangement, and its response is determined by the competition between strain gradient energy and constraint energy. When the foundation constraint is strong, macroscopic deformation is suppressed [18], leading to a relative enhancement of higher-order strain gradients. This effect potentially provides an additional driving force that excites the phason field, partially offsetting the suppression induced by size hardening and thus giving rise to a non-monotonic response.

**Figure 10:**
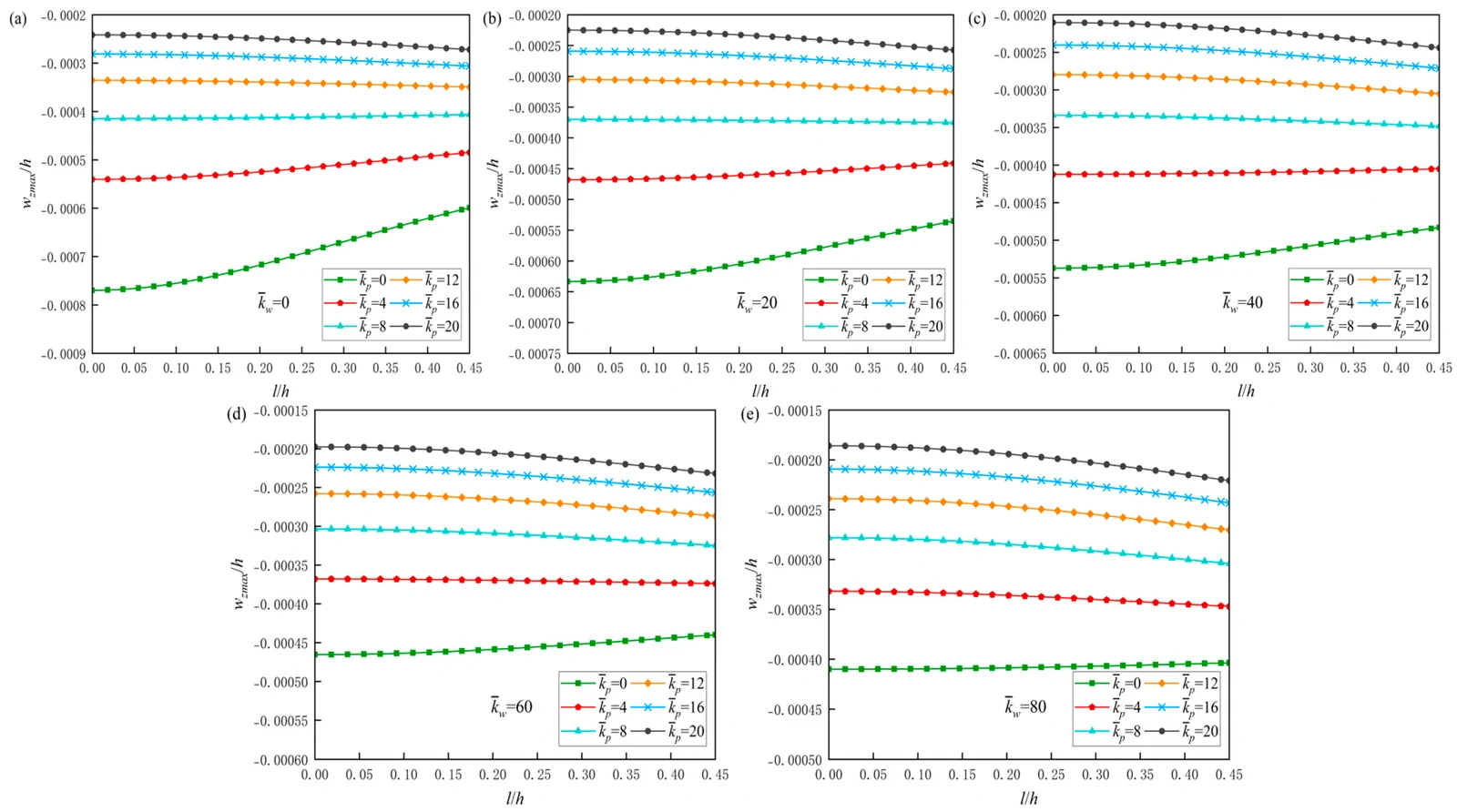
Variation in the maximum mid-span phason deflection with *l*/*h* and Pasternak shear stiffness under different Winkler spring stiffnesses: (**a**) ${\bar{k}}_{w}=0$; (**b**) ${\bar{k}}_{w}=20$; (**c**) ${\bar{k}}_{w}=40$; (**d**) ${\bar{k}}_{w}=60$; (**e**) ${\bar{k}}_{w}=80$.

Although Figure 9 has demonstrated that the overall constraint of the elastic foundation markedly alters the response trend of the phason field to the size effect, the independent contributions of the Winkler springs and the Pasternak shear layer remain unclear. The mechanical pathways of the two foundation parameters are fundamentally different: the Winkler springs provide a normal constraint proportional to the deflection, directly suppressing the displacement amplitude of the phonon field; the Pasternak shear layer participates in the rotational equilibrium of the phonon field through its in-plane shear stiffness, thereby modifying the curvature distribution. Since the phason field is driven by the deformation gradient of the phonon field via the phonon–phason coupling terms, the indirect regulatory effects of the two constraints on the phason field may differ significantly. Such a systematic distinction has not been reported in the existing literature. To systematically reveal the independent effects and the synergistic interplay of the two foundation parameters, Figure 10 presents the curves of the mid-span phason deflection versus the length scale parameter under five Winkler spring stiffnesses (${\bar{k}}_{w}=0,20,40,60,80$), with each ${\bar{k}}_{w}$ value containing six curves corresponding to different Pasternak shear stiffnesses (${\bar{k}}_{p}=0,4,8,12,16,20$).

From Figure 10, it is first observed that the mid-span phason deflection decreases globally as both foundation stiffnesses increase. Furthermore, increasing the Pasternak shear stiffness ${\bar{k}}_{p}$ not only suppresses the overall deflection but also triggers a qualitative transition in the mid-span phason response once a critical value is exceeded. Taking Figure 10a as an example: when ${\bar{k}}_{p}$= 0 and 4, the mid-span deflection decreases monotonically with increasing length scale parameter *l*/*h*. At ${\bar{k}}_{p}=8$, the curve becomes nearly flat, and the size sensitivity at the large-scale end virtually disappears. For ${\bar{k}}_{p}\geq 12$, a distinct “first decrease then increase” pattern emerges. The appearance of this rebound indicates that the Pasternak shear layer, while providing constraint, simultaneously opens an additional driving pathway for the phason field by reshaping the curvature distribution of the phonon field. This pathway is progressively enhanced once the size-induced stiffening reaches a certain level, thereby reversing the direction of the deflection variation. It can also be observed that the threshold value of ${\bar{k}}_{p}$ required to trigger this qualitative transition systematically decreases with increasing ${\bar{k}}_{w}$. This implies that the Winkler springs lower the threshold at which the Pasternak shear layer induces the non-monotonic response. Revealing a synergistic effect between the two foundation parameters on the morphological transition.

**Figure 11:**
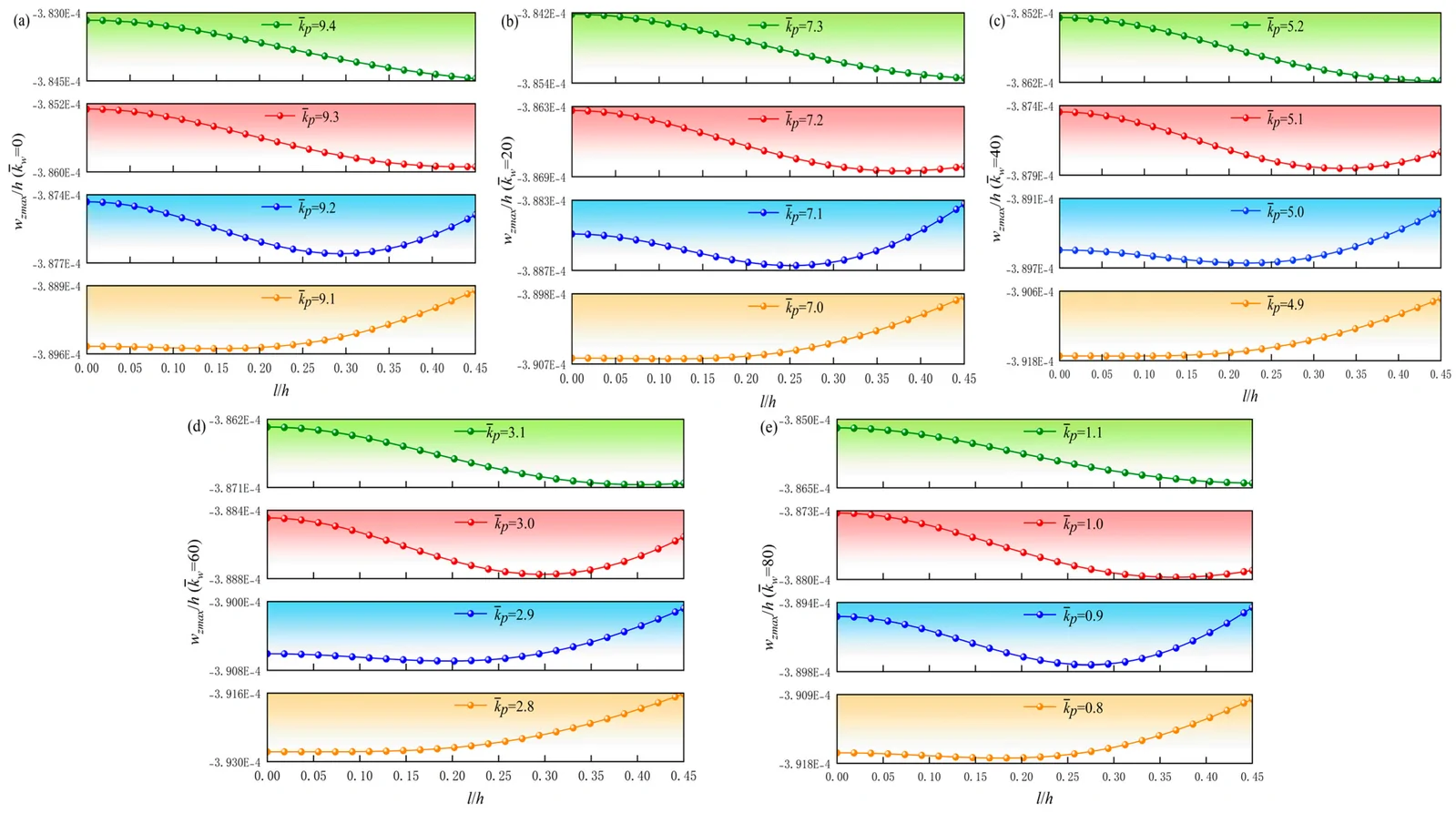
Fine-scan curves in the vicinity of the critical Pasternak shear stiffness under various Winkler spring stiffnesses: (**a**) ${\bar{k}}_{w}=0$; (**b**) ${\bar{k}}_{w}=20$; (**c**) ${\bar{k}}_{w}=40$; (**d**) ${\bar{k}}_{w}=60$; (**e**) ${\bar{k}}_{w}=80$.

The coarse-scan analysis in Figure 10 has shown that the threshold of ${\bar{k}}_{p}$ for the reversal of size sensitivity in the phason field systematically decreases with increasing ${\bar{k}}_{w}$. However, limited by the parameter step size (${\bar{k}}_{p}=4$), the precise critical transition interval and its evolution remain unclear. To address this, Figure 11 presents a fine scan of ${\bar{k}}_{p}$ (step size 0.1) around the critical interval at each ${\bar{k}}_{w}$ level, in order to capture the detailed characteristics of the transition.

Figure 11 reveals a key detail that the coarse scan failed to capture: at each ${\bar{k}}_{w}$ level, the reversal of size sensitivity does not occur at a single critical point, but instead passes through a transitional regime characterized by “first an increase, then a decrease.” Within the transition interval, the phason mid-span deflection first exhibits an anomalous rise in the small *l*/*h* range, reaches a peak, and then resumes a decreasing trend in the large *l*/*h* range, forming an overall “increase-then-decrease” transitional morphology. As ${\bar{k}}_{p}$ further increases and crosses the critical interval, the curve eventually transitions to a monotonic rise. The existence of a critical interval indicates that this transition is not gradual; rather, two competing mechanisms complete a handover of dominance within a narrow stiffness band. A cross-comparison across different ${\bar{k}}_{w}$ values further shows that the Winkler springs, through their normal constraint, concentrate the deformation towards the boundaries and thereby indirectly enhance the transmission efficiency of the Pasternak shear layer, causing the critical interval to undergo a systematic forward shift with increasing ${\bar{k}}_{w}$. The coarse and fine scans described above provide a complete picture of the reversal of the phason size sensitivity, but they do not by themselves reveal the underlying energy mechanism responsible for this transition. It should be emphasized that the critical Pasternak stiffness identified in this study is an apparent transition value obtained under the specific conditions of the present analysis, namely the Al-Ni-Co quasicrystal material parameters, the assumed beam geometry *L*/*h* = 20, *b*/*h* = 2, simply supported boundary conditions, and the chosen scanning ranges and step sizes for ${\bar{k}}_{w},{\bar{k}}_{p}$, and *l*/*h*. Since the transition is detected from a finite set of numerical points, the critical value should be interpreted as a transition band rather than a universal material constant. Nevertheless, the qualitative features of the transition—the existence of a non-monotonic phason response and the decrease in the required Pasternak stiffness with increasing Winkler stiffness—are expected to remain valid within the present modified couple-stress and Winkler–Pasternak framework, although the quantitative thresholds may shift if the beam geometry, boundary conditions, or material constants are changed. Near the critical interval, the size effect and the foundation constraint simultaneously alter the phonon deformation state, and the phason displacement is affected through the phonon–phason coupling terms. It is therefore necessary to decompose the energy components closely related to the phason response and to examine their variation with *l*/*h*, so as to clarify the relative roles of different mechanisms during the transition.

The total strain energy in Equation (7) contains not only the phonon elastic strain energy but also the phason elastic strain energy, the phonon–phason coupling energy, the couple-stress energy, and the foundation strain energy. Among these, the phonon elastic strain energy dominates the total energy and varies monotonically with *l*/*h*. Including it in the normalization would strongly compress the percentages of the other relevant terms and obscure their variations. The phason elastic strain energy, on the other hand, is a passive response after the phason field is excited and does not explain why the phonon deformation changes.

In the present static bending problem, the phason displacement is not subjected to an external force directly; rather, it is driven by the phonon shear deformation through the phonon–phason coupling term. The key factor controlling the phason response is therefore which mechanism dominates the phonon deformation: the microscale hardening induced by the couple stress or the elastic constraint provided by the foundation. Accordingly, we extract the couple-stress energy *U_couple_*, the foundation strain energy *U_foundation_*, and the phonon–phason coupling energy *U_coupling_*, and define the competing subset

(31) $$U_{compete}=\left|U_{couple}\right|+\left|U_{foundation}\right|+\left|U_{coupling}\right|$$

The percentage of each component within this subset is then analyzed. Absolute values are used to avoid sign ambiguity arising from the coupling term. It should be noted that this energy-based quantification of the phason transition has not been reported in existing size-dependent quasicrystal beam models, where the non-monotonic phason response was either absent or interpreted only qualitatively.

**Figure 12:**
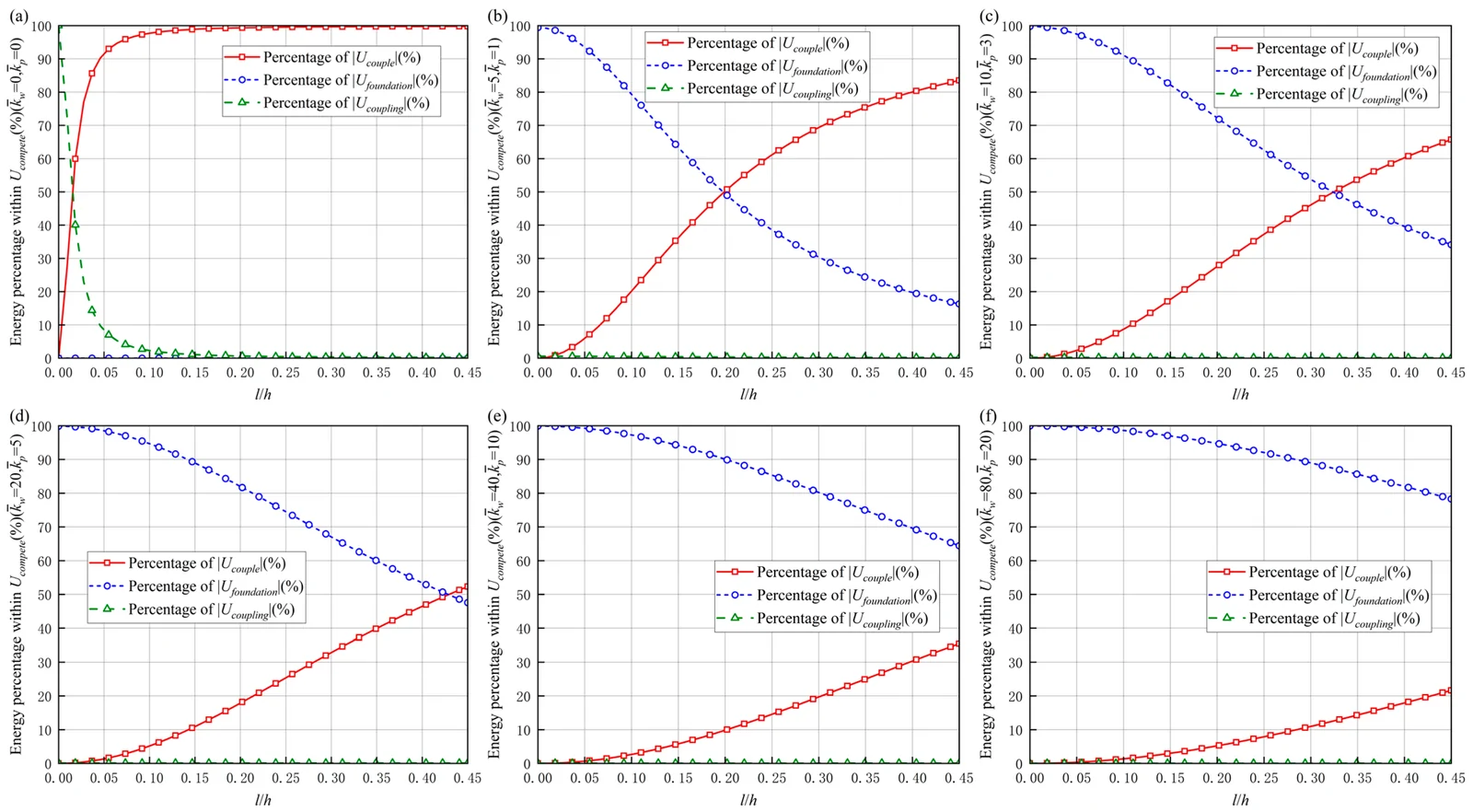
Energy-based decomposition for the phason transition. Percentages of U_couple_, U_foundation_, and U_coupling_ within U_compete_ versus l/h. (**a**) (${\bar{k}}_{w},{\bar{k}}_{p}$) = (0, 0); (**b**) (${\bar{k}}_{w},{\bar{k}}_{p}$) = (5, 1); (**c**) (${\bar{k}}_{w},{\bar{k}}_{p}$) = (10, 3); (**d**) (${\bar{k}}_{w},{\bar{k}}_{p}$) = (20, 5); (**e**) (${\bar{k}}_{w},{\bar{k}}_{p}$) = (40, 10); (**f**) (${\bar{k}}_{w},{\bar{k}}_{p}$) = (80, 20). In each subfigure, the three curves denote U_couple_, U_foundation_, and U_coupling_, respectively; the vertical axis is percentage (%).

Figure 12 shows the variations in these three energy percentages with *l*/*h* for six representative foundation parameter combinations. As *l*/*h* increases, the couple-stress energy percentage increases monotonically, while the foundation energy percentage decreases correspondingly. The phonon–phason coupling energy percentage remains negligibly small, typically below 1%, and it becomes noticeable only in the absence of a foundation and for very weak size effects. When the foundation stiffness is relatively large, the foundation energy percentage remains higher than the couple-stress energy percentage over most of the considered *l*/*h* range, and no clear crossing between the two curves is observed. Although the foundation energy percentage decreases with increasing *l*/*h*, it remains at a relatively high level. This indicates that a strong foundation constraint can maintain its dominant share within the competing subset to a certain extent, rather than being completely replaced by the couple-stress energy. These features suggest that although the phonon–phason coupling term is necessary for transmitting the phonon deformation to the phason field, its energy magnitude is not the main factor controlling the non-monotonic transition. The transition is primarily associated with the relative change between the couple-stress energy and the foundation energy. Therefore, in the following analysis, the coupling energy can be reasonably excluded from the normalization, and attention can be focused on the competition between the foundation energy and the couple-stress energy.

To quantify the above competition, we introduce the energy partition ratio:

(32) $$R=\frac{\left|U_{foundation}\right|}{\left|U_{foundation}\right|+\left|U_{couple}\right|}$$

**Figure 13:**
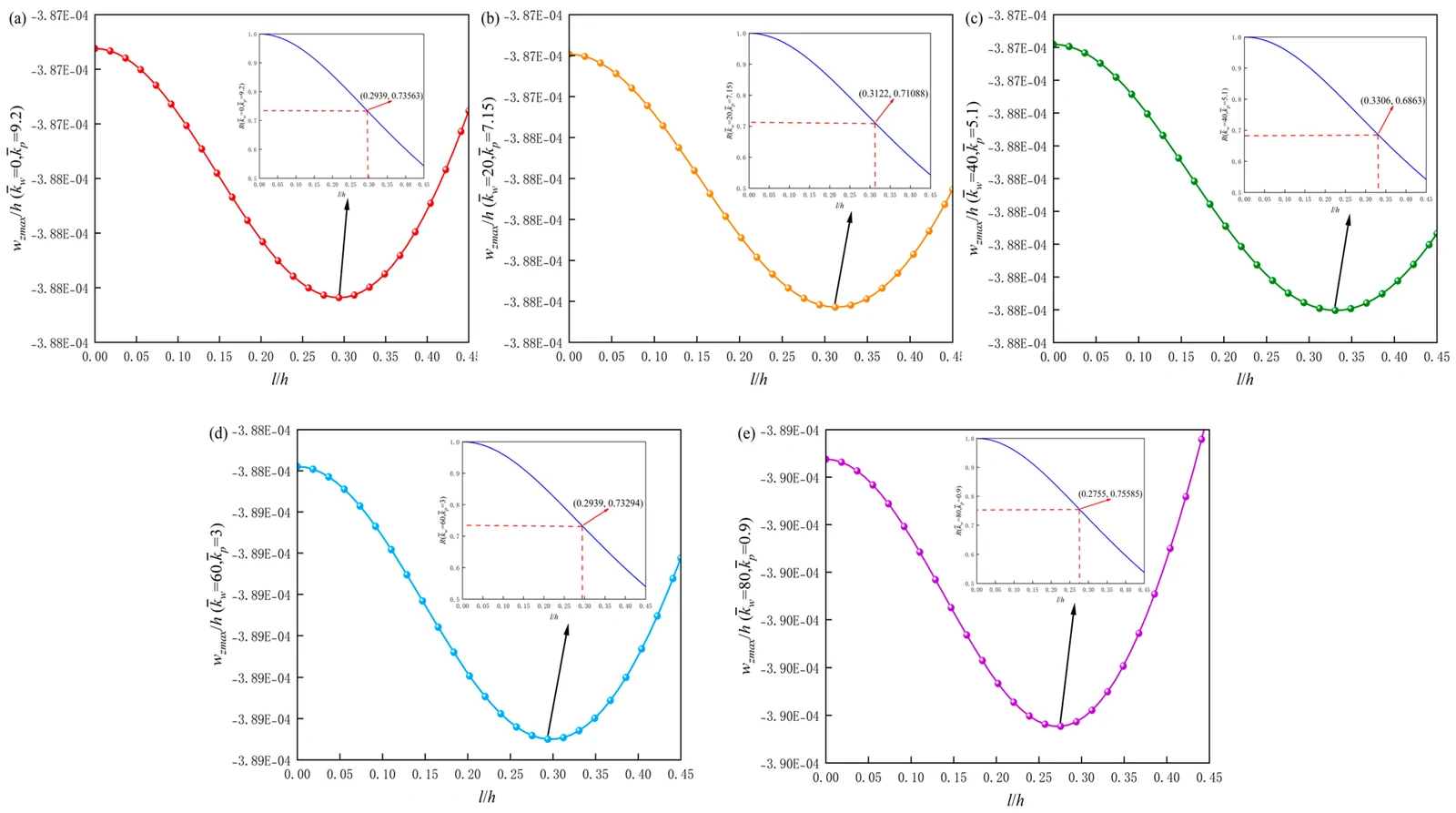
Critical energy partition controlling the phason transition. Correspondence between the inflection points of the phason mid-span deflection and the energy partition ratio *R* for different foundation parameter combinations. The arrows indicate the *R* values at the same *l*/*h* positions as the inflection points.

This ratio represents the relative weight of the foundation constraint in the competing pair of foundation constraint and couple-stress hardening. A value of *R* close to 1 indicates that the foundation constraint dominates, whereas a value close to 0 indicates that couple-stress hardening dominates. If the non-monotonic phason response is indeed governed by the competition between these two mechanisms, then the inflection points obtained under different foundation parameter combinations should correspond to similar *R* values. Figure 13 shows the positions of the inflection points on the *R*–*l*/*h* curves to examine this expectation.

Figure 13 projects the inflection points of the phason deflection onto the corresponding *R* curves. The results show that, for the cases in which an inflection point appears, the *R*–*l*/*h* curves exhibit very similar overall trends. This quantitative consistency provides new evidence that the phason transition is physically driven rather than a numerical artifact. Although the inflection points occur at different *l*/*h* positions depending on the foundation parameters, the corresponding *R* values fall within a narrow band of 0.68∼0.75. This indicates that the appearance of the non-monotonic phason response is not directly determined by the absolute magnitude of the size-effect strength, but rather by the internal state characterized by the energy partition ratio. When *R* lies within 0.68∼0.75, the foundation constraint is still dominant, but the couple-stress hardening has already reached a considerable level. Under this condition, the Pasternak shear layer can still effectively regulate the phonon shear deformation, while the size effect is strong enough to locally modify the deformation mode, thereby triggering the rebound of the phason displacement. Once the couple-stress energy becomes completely dominant, the phonon shear deformation is suppressed by the size effect, and the non-monotonic response disappears.

This finding is also consistent with the previously observed synergistic effect between the Winkler and Pasternak parameters. The Winkler springs suppress the transverse deflection and thereby reduce the Pasternak shear stiffness required to maintain the same critical energy partition, so that different foundation combinations can reach a similar *R* value at their respective inflection points.

### 3.3. Analysis of Normalized Contact Stiffness

The normalized contact stiffness of the elastic foundation is examined by incorporating the influence of both the beam size effect and the foundation stiffness on the contact behavior of the beam system [46]. This stiffness is defined as

(33) $$\bar{K}=\frac{u_{z,x=L/2}}{u_{z,x=L/2}^{l}},$$

where $u_{z,x=L/2}$ is the central displacement (*x* = *L*/2, *y* = 0, *z* = 0) of the classical simply supported beam under a uniformly distributed load and elastic foundation, and $u_{z,x=L/2}^{l}$ is the central displacement (*x* = *L*/2, *y* = 0, *z* = 0) of the present simply supported beam that accounts for the size effect, subjected to the same load and supported by the same elastic foundation. When the Pasternak shear layer stiffness $k_{p}$ vanishes, the foundation model reduces to the Winkler foundation model.

**Figure 14:**
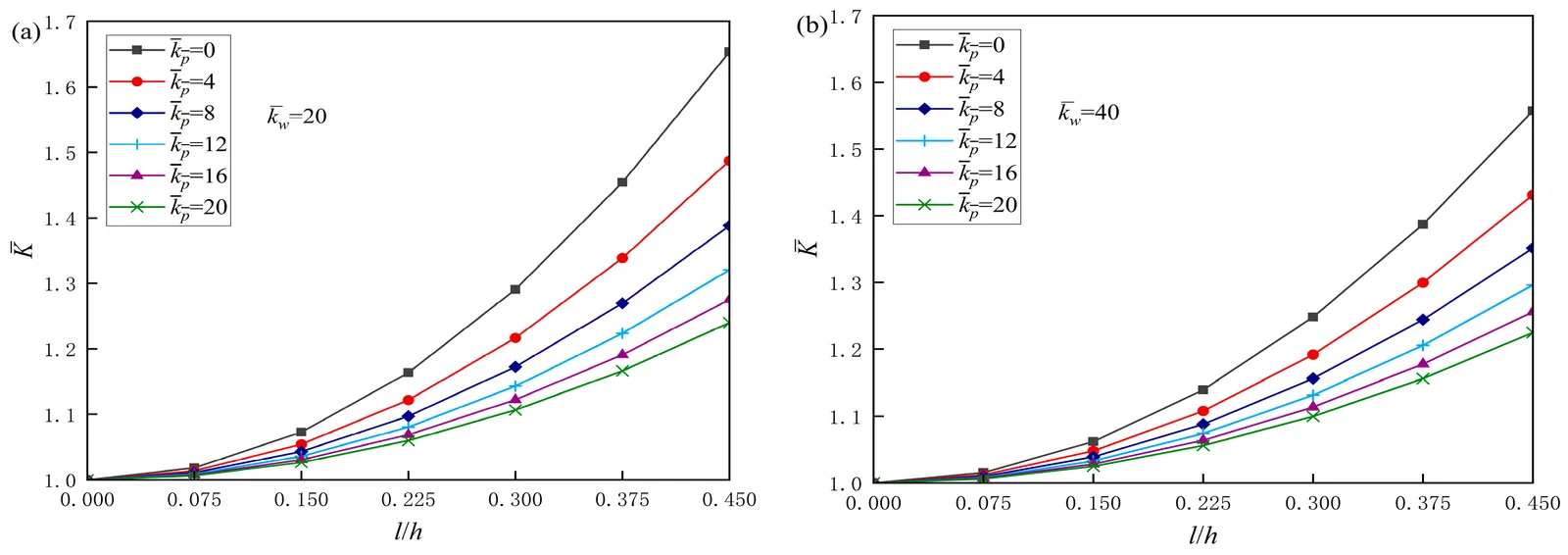

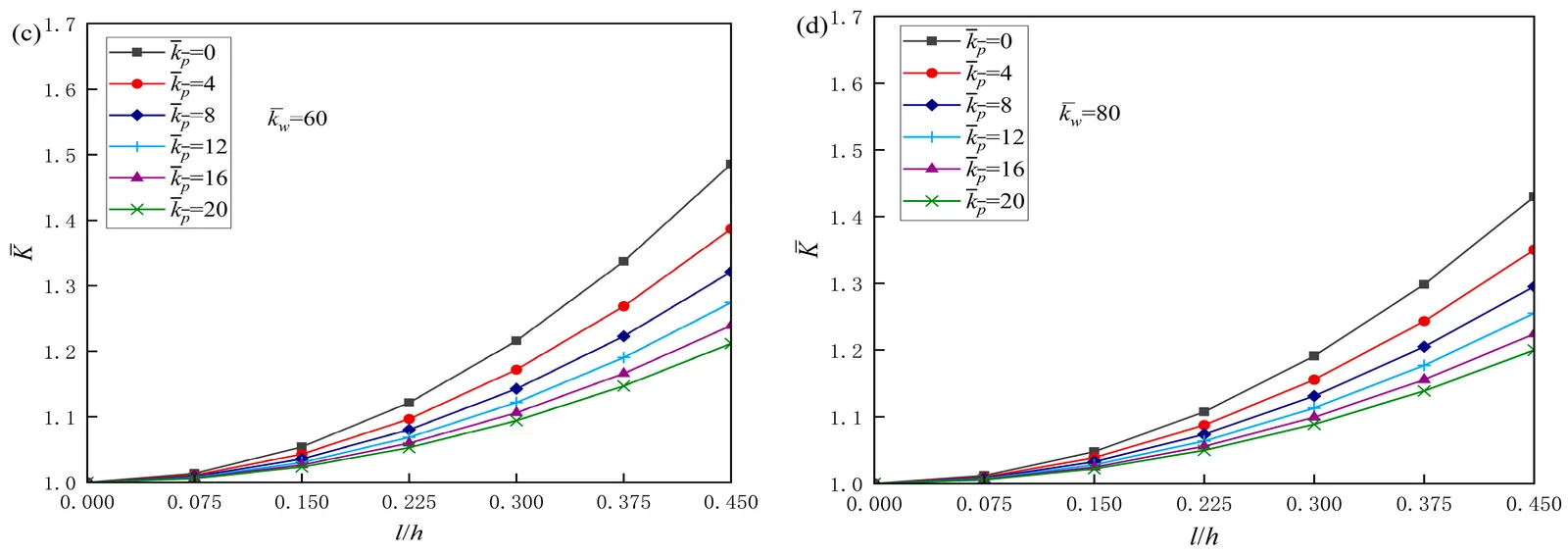
Variation in the normalized system contact stiffness under different ${\bar{k}}_{w}$ and ${\bar{k}}_{p}$: (**a**) ${\bar{k}}_{w}=20$; (**b**) ${\bar{k}}_{w}=40$, (**c**) ${\bar{k}}_{w}=60$; (**d**) ${\bar{k}}_{w}=80$.

Figure 14 shows the influence of the material length scale parameter and the foundation stiffness parameters on the normalized system contact stiffness $\bar{K}$ for a simply supported 1D hexagonal piezoelectric quasicrystal microbeam. The normalized stiffness $\bar{K}$ rises markedly as the length scale parameter *l* (or *l*/*h*) increases, demonstrating the size hardening effect. The normalized contact stiffness reaches its maximum at ${\bar{k}}_{p}=0$, which corresponds to the Winkler foundation model. As ${\bar{k}}_{w}$ increases, the stiffness stabilizes, suggesting that a stiffer foundation weakens the sensitivity to the size effect. In contrast, the normalized contact stiffness ${\bar{k}}_{p}$ tends to decrease overall as $\bar{K}$ increases, indicating that shear coupling effectively distributes the load and suppresses the excessive hardening induced by the size effect.

### 3.4. Free Vibration Response

**Figure 15:**
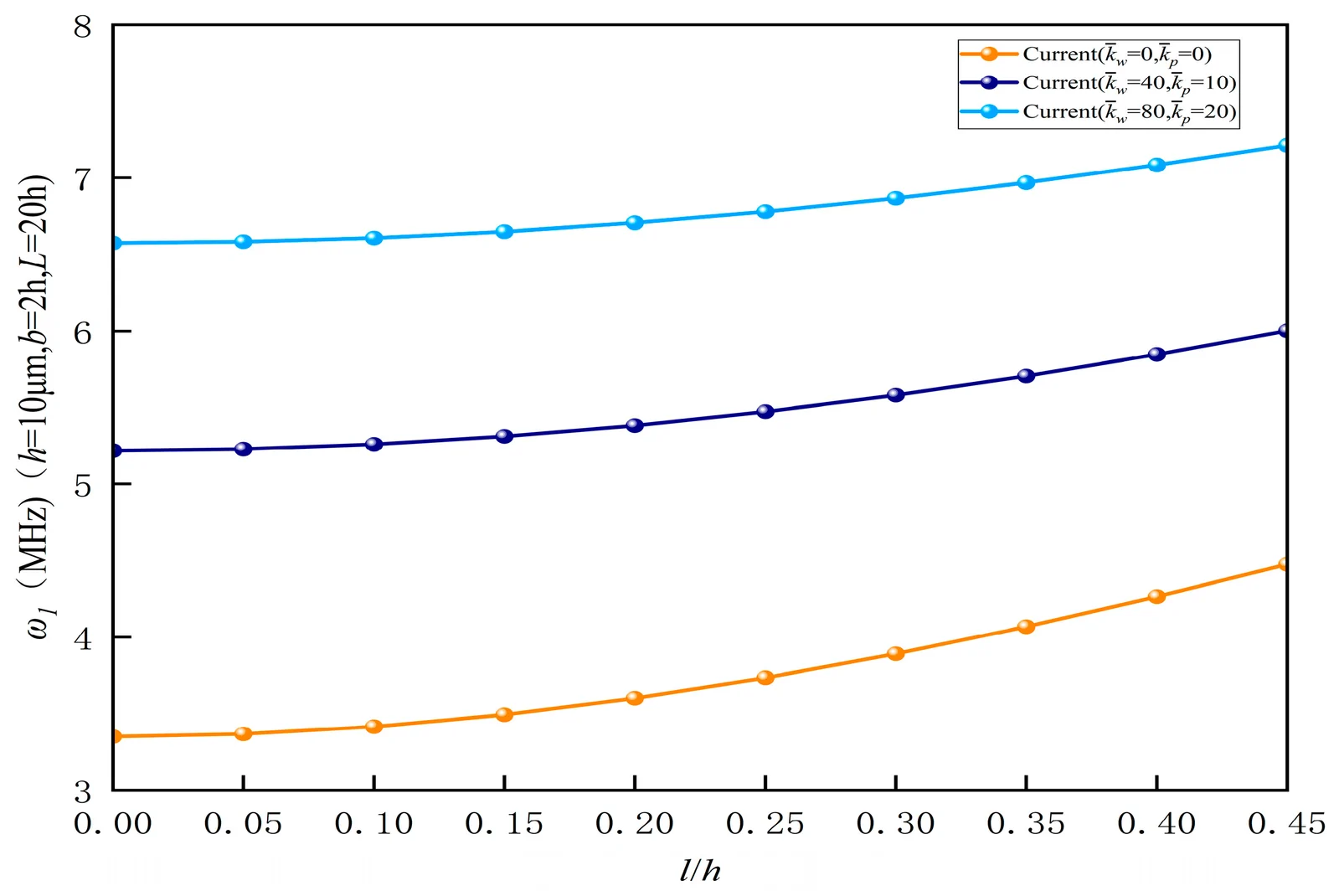
Variation in the first natural frequency with *l*/*h* for a simply supported 1D hexagonal piezoelectric quasicrystal Timoshenko microbeam with *L*/*h* = 20 under three representative foundation parameter combinations.

To examine the combined influence of the material length scale parameter and the elastic foundation on the vibration behavior, the first natural frequency of a simply supported 1D hexagonal piezoelectric quasicrystal Timoshenko microbeam with *L*/*h* = 20 is calculated for three representative foundation parameter combinations: (${\bar{k}}_{w},{\bar{k}}_{p}$) = (0,0), (40,10) and (80,20). The corresponding results are shown in Figure 15.

As shown in Figure 15, under all three foundation conditions, the first natural frequency varies smoothly and monotonically with *l*/*h* over the entire investigated interval, and no abrupt changes or singularities are observed. At a fixed *l*/*h*, the foundation stiffness clearly raises the natural frequency, and this enhancement becomes more pronounced as ${\bar{k}}_{w}$ and ${\bar{k}}_{p}$ increase. This indicates that the size effect and the foundation effect jointly govern the free vibration response. The monotonic behavior also confirms that the chosen range is physically reasonable, and that the free vibration predictions are not unduly sensitive to the exact value of the material length scale parameter. The subsequent results are therefore presented for the selected discrete values of *l*/*h* within this range.

**Figure 16:**
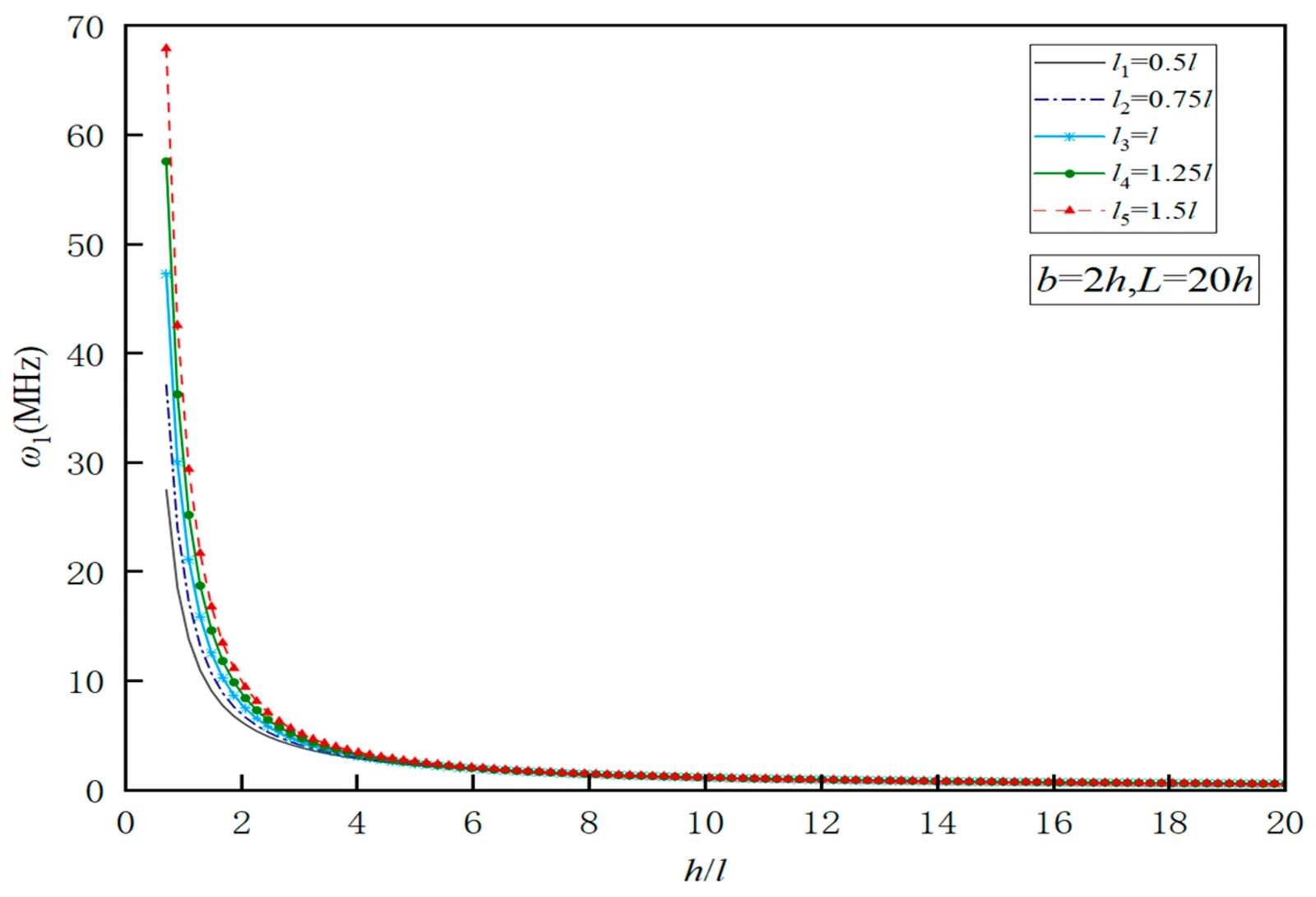
Influence of the length scale parameter *l* on the first natural frequency *ω*_1_ of the 1D hexagonal piezoelectric quasicrystal Timoshenko beams ($k_{w}=k_{p}=0$).

To examine how the material length scale parameter influences the first natural frequency, the parameter *l* is taken as 3 μm (corresponding to *l*/*h* = 0.3 when *h* =10 μm as in Section 2) and scaled accordingly. As shown in Figure 16, for a fixed *h*/*L* ratio, the first natural frequency of the 1D hexagonal piezoelectric quasicrystal increases with increasing *l*. This effect diminishes sharply at large *h*/*L* (macroscopic size), indicating that the size effect exhibits a pronounced scale dependence.

**Figure 17:**
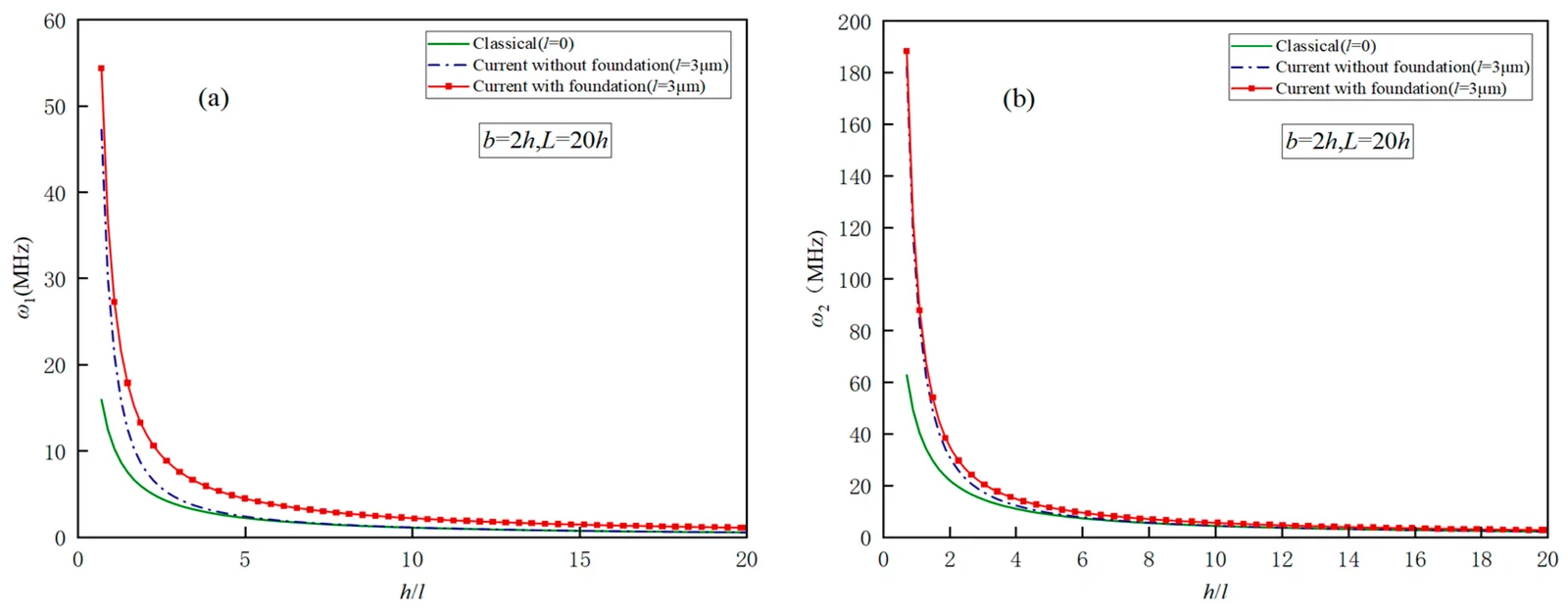
(**a**) First natural frequency $\omega_{1}$ and (**b**) second natural frequency $\omega_{2}$ versus beam thickness.

Figure 17 shows the variations in the first two natural frequencies predicted by the classical beam model, the present beam model without the foundation (including size effects), and the present beam model incorporating a Winkler–Pasternak foundation, as functions of the beam thickness. The results show that the first two natural frequencies obtained from the present beam model are consistently higher than those predicted by the classical beam model. Incorporating the Winkler–Pasternak foundation further elevates the predicted frequencies. These findings indicate that both the size effects and the foundation effect contribute to increasing the beam’s natural frequencies. Such effects are most pronounced for small beam thickness (i.e., small *h*/*L*), whereas as the thickness increases (large *h*/*L*), their influences diminish rapidly, and the predictions from all models tend to coincide.

**Figure 18:**
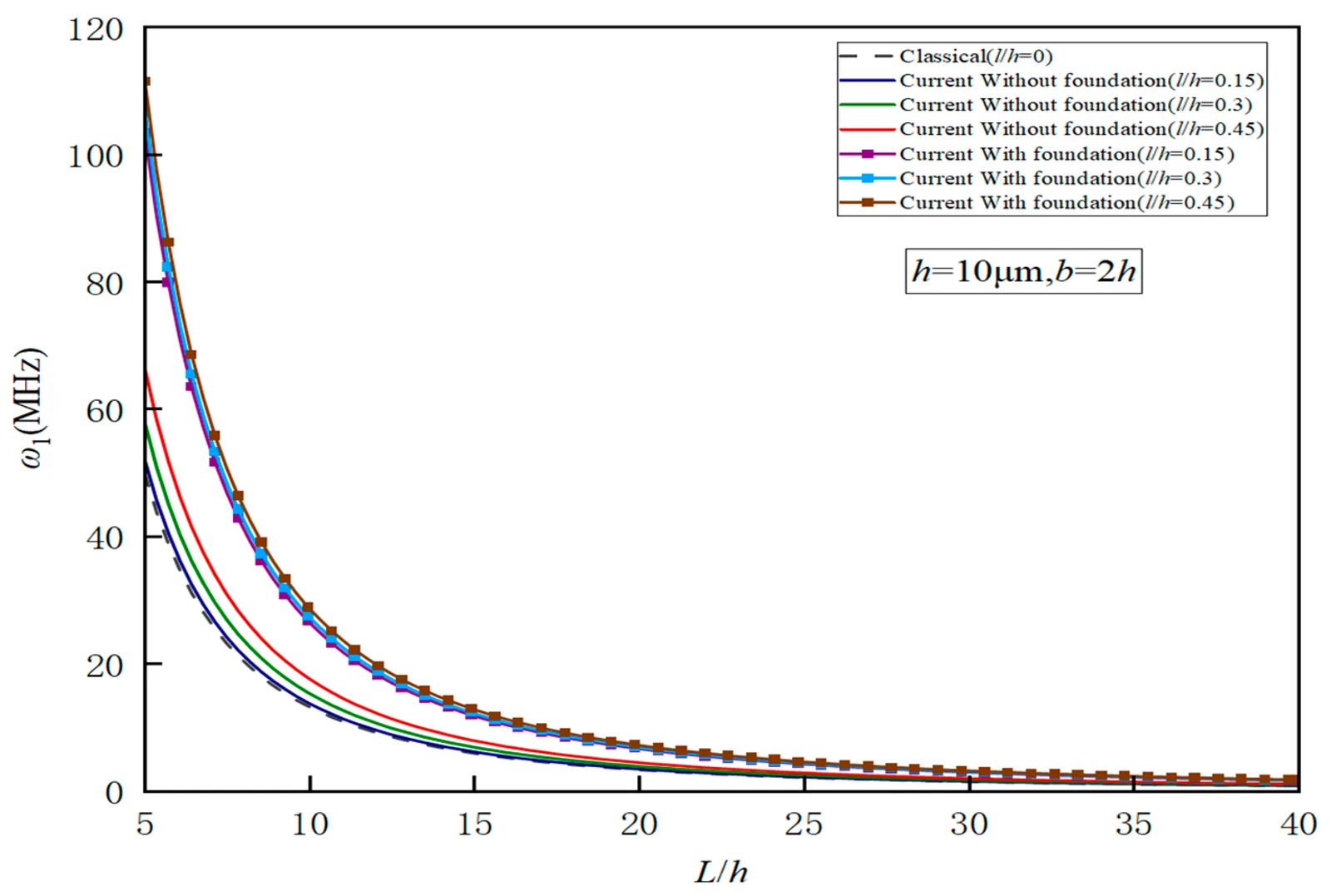
First natural frequency $\omega_{1}$ versus span-to-height ratio.

Figure 18 shows how the first natural frequency varies with the span-to-height ratio for the classical beam model, the present beam model without foundation, and the present beam model with foundation, each at different values of the length scale parameter. The natural frequency decreases as the span-to-height ratio *L*/*h* increases. Both the size effect and foundation effect contribute to raising the natural frequency. Notably, these enhancement effects are more pronounced for short beams.

**Figure 19:**
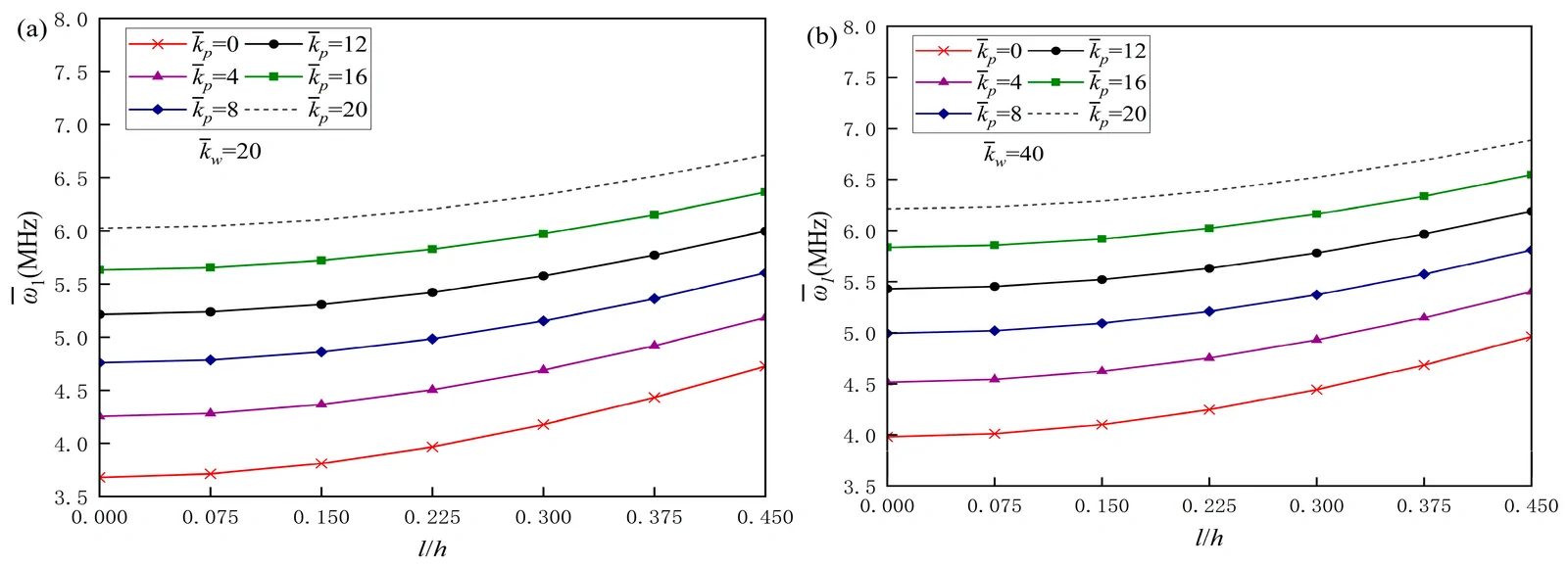

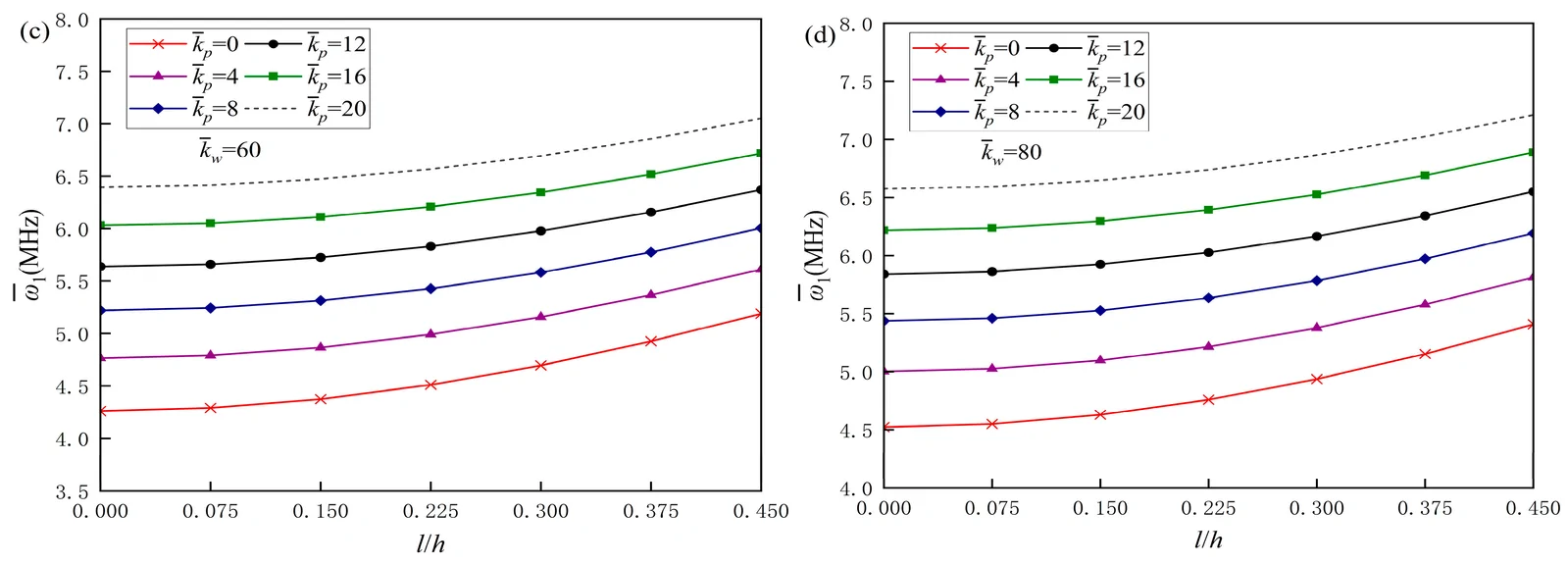
Variation in the first natural frequency $\omega_{1}$ under different ${\bar{k}}_{w}$ and ${\bar{k}}_{p}$: (**a**) ${\bar{k}}_{w}=20$; (**b**) ${\bar{k}}_{w}=40$; (**c**) ${\bar{k}}_{w}=60$; (**d**) ${\bar{k}}_{w}=80$.

Figure 19 plots the first natural frequency against the length scale parameter ratio (*l*/*h*) for different Winkler spring stiffness ($k_{w}$) and Pasternak shear layer stiffness ($k_{p}$). The figure systematically reveals the coupled effects of the foundation parameters and size effect on the natural frequency. All curves increase monotonically as both *l*/*h* and the foundation stiffness parameters ($k_{w},k_{p}$) increase. The Winkler stiffness $k_{w}$ dominates the enhancement of the natural frequency. The influence of the foundation is strongest when the size effect remains weak, i.e., at small *l*/*h*. As the size effect becomes strong (large *l*/*h*), the relative influence of the foundation effect decreases, but it still plays a key role.

## 4. Conclusions

A new model for 1D hexagonal piezoelectric quasicrystal Timoshenko microbeams is developed using Hamilton’s principle, the extended modified couple stress theory, and the Winkler–Pasternak two-parameter elastic foundation model. The governing equations and boundary conditions simultaneously incorporate size effects, piezoelectric coupling, and foundation support. The proposed model not only captures the mechanical behavior under classical conditions but also effectively predicts the influences of size effects and foundation stiffness on the structural response, providing a theoretical reference for the multi-physics analysis of quasicrystal microbeams. Through static bending and free vibration analyses of simply supported beams, the coupled effects of the material size effect and foundation stiffness on the mechanical behavior of beam structures are examined. Compared with existing size-dependent quasicrystal beam formulations, the present work differs mainly in three aspects: the simultaneous incorporation of a two-parameter Winkler–Pasternak foundation and piezoelectric coupling in a Timoshenko microbeam, the introduction of a normalized system contact stiffness to quantify the size–foundation interaction, and the identification of a non-monotonic phason response controlled by a critical energy partition ratio. The following conclusions are reached.

1. The size effect significantly enhances the macroscopic stiffness of the beam and suppresses the piezoelectric output. After the size effect is taken into account, the deflection, rotation angle, axial normal stress, and electric potential of the beam are significantly reduced and decrease further with increasing material length scale parameter *l*, whereas the natural frequencies of free vibration are increased accordingly. This “size hardening” effect is particularly pronounced for beams of small thickness.

2. The Winkler–Pasternak foundation further suppresses deformation and exerts markedly different influences on the phonon and phason fields. While the phonon response decreases monotonically with increasing foundation stiffness, the phason field exhibits a non-monotonic size sensitivity that can be reversed once the Pasternak stiffness exceeds a critical value. This threshold decreases systematically with increasing Winkler stiffness, giving rise to a synergistic effect between the two foundation parameters; fine scanning further shows that the reversal occurs through a narrow transition band rather than at a single critical point. Energy decomposition indicates that the phonon–phason coupling energy remains below 1% within the competing subset, whereas the inflection points of the phason deflection correspond to a narrow band of the energy partition ratio *R* between the foundation and couple-stress energies (approximately0.68∼0.75). This confirms that the transition is governed primarily by the relative energy competition rather than by the coupling energy itself, which differs from existing size-dependent quasicrystal beam models where the phason response generally remains monotonic without the two-parameter foundation.

3. The normalized contact stiffness analysis reveals the regulating effect of the foundation parameters. The size effect causes the normalized system stiffness to rise, exhibiting obvious hardening behavior in the contact stiffness. As the material length scale parameter increases, the stiffness curve tends to stabilize, indicating that a strong foundation constraint weakens the sensitivity to the size effect. The normalized contact stiffness ($\bar{K}$) reaches its maximum in the pure Winkler foundation model (${\bar{k}}_{p}=0$), and it decreases gradually with the introduction of the Pasternak shear layer (${\bar{k}}_{p}>0$), showing that shear coupling can effectively alleviate local stiffness concentration. This finding provides a possible reference for evaluating the influence of foundation parameters.

4. The free vibration characteristics are jointly governed by the size effect and the foundation effect. The influence of the size effect on the natural frequency is particularly significant when the beam thickness and span-to-height ratio are small. The contribution of the foundation stiffness (especially the Winkler stiffness) to the frequency dominates when the size effect is weak. For the preliminary theoretical analysis of microbeam structures, the coupling between size effects and foundation support should be considered. The present results may also provide a theoretical reference for the mechanical analysis of quasicrystal films or coatings on elastic substrates in materials engineering, although direct design applications require experimental calibration.

It should be mentioned that the present analysis adopts idealized assumptions, including an open-circuit electrical condition and idealized material/foundation parameters. The predicted quasi-static electric potential therefore reflects the intrinsic piezoelectric response; in high-frequency or electrically insulated configurations, charge redistribution effects in conductive media are expected to be less pronounced, and the present results may still offer useful theoretical insight. The reported critical Pasternak stiffness intervals are obtained for the specific Al-Ni-Co material and geometry considered here, and their quantitative values should be regarded as apparent transition bands rather than universal material constants. Nevertheless, the qualitative mechanisms revealed by this analysis—especially the non-monotonic phason response and the synergistic shift in the critical Pasternak stiffness with the Winkler stiffness—are expected to remain valid within the present modified couple-stress and Winkler–Pasternak framework.

## Figures and Tables

**Table 1:** A comparison of the first natural frequency for free vibration of isotropic beams under different foundation stiffnesses.

Foundation Parameters		*h*/*L* = 1/120		*h*/*L* = 1/15		*h*/*L* = 1/5	
${\bar{k}}_{w}$	${\bar{k}}_{p}$ **/π^2^**	Present	SSDQM	Present	SSDQM	Present	SSDQM
0	0	3.14642	3.14143	3.13207	3.13024	3.08325	3.04799
	0.5	3.46258	3.47659	3.46685	3.46671	3.40698	3.39458
	1.0	3.73612	3.73587	3.72673	3.72656	3.66440	3.65802
	2.5	4.29696	4.29686	4.28812	4.28809	4.21916	4.21834
10^2^	0	3.75318	3.74823	3.74707	3.73894	3.67146	3.67050
	0.5	3.96891	3.96067	3.95303	3.95168	3.88923	3.88397
	1.0	4.14705	4.14356	4.13629	4.13471	4.06856	4.06636
	2.5	4.58713	4.58226	4.58151	4.57347	4.50301	4.49913

**Table 2:** A comparison of the first three natural frequencies of simply supported 1D hexagonal piezoelectric quasicrystal Timoshenko microbeams.

*L*/*h*	*l*/*h*	Present (n = 1)	Ref. [40] (n = 1)	Present (n = 2)	Ref. [40] (n = 2)	Present (n = 3)	Ref. [40] (n = 3)
20	0	2.8581	2.8700	11.3259	11.3299	25.7661	24.9647
	0.1	2.9318	2.9403	11.5522	11.6054	25.5691	25.5657
	0.2	3.0696	3.1416	12.3064	12.3914	27.2647	27.2715
30	0	2.8486	2.8772	11.4289	11.4404	25.5037	25.4922
	0.1	2.9256	2.9478	11.7168	11.7202	26.1158	26.1122
	0.2	3.1103	3.1500	12.5050	12.5202	27.7956	27.8808

**Table 3:** Material parameters of Al-Ni-Co quasicrystal (QC1).

***C*_11_**	***C*_12_**	***C*_13_**	***C*_33_**	***C*_44_**	***C*_66_**	***K*_1_**	***K*_2_**	***R*_1_**	***R*_2_**
234.33	57.41	66.63	232.22	70.19	88.46	122	24	8.846	8.846
*R*_3_	*e*_31_	*e*_33_	*e*_15_	*d*_15_	*d*_33_	*λ*_11_	*λ*_33_	*ρ*	
8.846	−4.4	18.6	11.6	1.16	1.86	5	10	4.168	

## Data Availability

The original contributions presented in this study are included in the article. Further inquiries can be directed to the corresponding author.
